# A Bioorthogonally Applicable, Fluorogenic, Large Stokes-Shift Probe for Intracellular Super-Resolution Imaging of Proteins

**DOI:** 10.3390/biom10030397

**Published:** 2020-03-04

**Authors:** Evelin Németh, Gergely Knorr, Krisztina Németh, Péter Kele

**Affiliations:** Chemical Biology Research Group, Institute of Organic Chemistry, Research Centre for Natural Sciences, Magyar tudósok krt 2, H-1117 Budapest, Hungary; nemeth.evelin@ttk.hu (E.N.); knorrgergely@gmail.com (G.K.); nemeth.krisztina@ttk.hu (K.N.)

**Keywords:** fluorogenic, large Stokes-shift, bioorthogonal, super-resolution, STED microscopy, multicolor labeling

## Abstract

Herein, we present the synthesis and application of a fluorogenic, large Stokes-shift (>100 nm), bioorthogonally conjugatable, membrane-permeable tetrazine probe, which can be excited at common laser line 488 nm and detected at around 600 nm. The applied design enabled improved fluorogenicity in the orange/red emission range, thus efficient suppression of background and autofluorescence upon imaging biological samples. Moreover, unlike our previous advanced probes, it does not require the presence of special target platforms or microenvironments to achieve similar fluorogenicity and can be generally applied, e.g., on translationally bioorthogonalized proteins. Live-cell labeling schemes revealed that the fluorogenic probe is suitable for specific labeling of intracellular proteins, site-specifically modified with a cyclooctynylated, non-canonical amino acid, even under no-wash conditions. Furthermore, the probe was found to be applicable in stimulated emission depletion (STED) super-resolution microscopy imaging using a 660 nm depletion laser. Probably the most salient feature of this new probe is that the large Stokes-shift allows dual-color labeling schemes of cellular structures using distinct excitation and the same detection wavelengths for the combined probes, which circumvents chromatic aberration related problems.

## 1. Introduction

Fluorescent methods are distinguished amongst the techniques dedicated to the sensitive and accurate detection of cellular and subcellular events in vivo. Due to recent hardware developments in super-resolution microscopy, it is rather the lack of suitable probes, which is considered as the major limitation of further improvements [1,2]. Therefore, there is a clear need for advanced probes suitable for selective targeting and in vivo super-resolution imaging of intracellular structures. Such membrane permeable, improved probes should address challenges such as selective conjugatability, autofluorescence, and background fluorescence [3]. Autofluorescence can be overcome by carefully selecting fluorescent frameworks that are either excitable towards the red range of the spectrum or possess large Stokes-shifts. Suppression of background fluorescence of non-specifically bound probes, on the other hand, is more challenging and often requires extensive washing cycles following staining. An alternative approach to minimize background fluorescence becomes possible with the use of fluorogenic probes that possess quenched fluorescence until a specific reaction with their target [4,5,6]. With respect to selective conjugatability, several strategies have evolved lately as a result of recent advances in gene technology and chemical biology [7]. Bioorthogonal ligations [8] in combination with genetic code expansion (GCE) [9] technology, is probably the most powerful method for the selective and site-specific installation of virtually non-perturbing, synthetic probes onto the protein of interest (POI). Such an approach is used routinely in the labeling schemes of various proteins, in cellulo or in vivo [6,7,8,10,11,12].

**Scheme 1:**
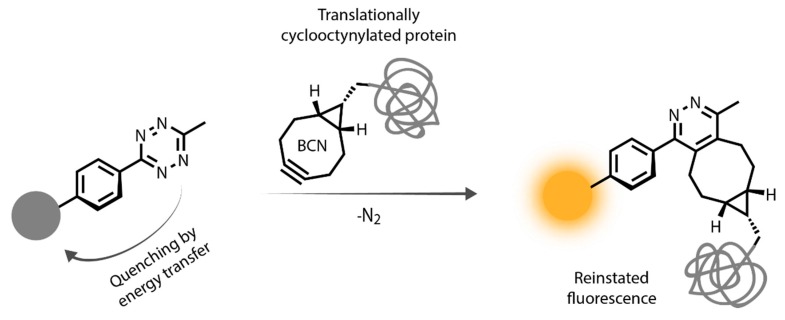
Representation of bioorthogonal labeling schemes involving tetrazine quenched fluorogenic probes and genetically bioorthogonalized proteins.

**Figure 1:**
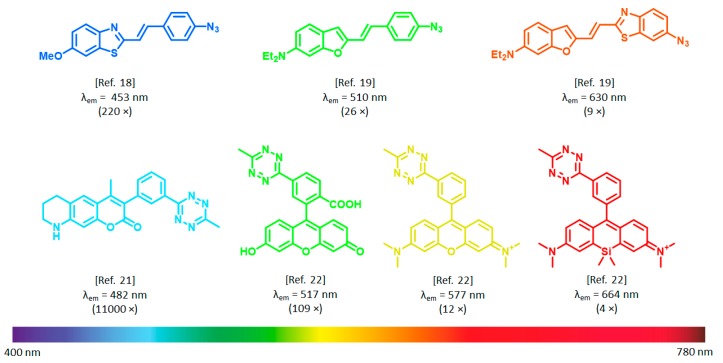
Examples for azide and tetrazine quenched bioorthogonally applicable fluorogenic probes (numbers in parentheses indicate fluorogenicites).

In terms of reaction kinetics, biocompatibility, synthetic accessibility, and compatibility with GCE, strain promoted azide–alkyne cycloaddition (SPAAC) of azides and strained alkynes [13] and the inverse electron demand Diels–Alder (IEDDA) reaction of tetrazines and strained alkenes/alkynes [14] are the most robust bioorthogonal reactions. Besides, as we and others have recently demonstrated, functional groups of the SPAAC and IEDDA reactions, i.e., the azide and the tetrazine moieties, are able to modulate (i.e., quench) fluorescence by various mechanisms (e.g., energy transfer [15,16,17], rotation-induced relaxation [18,19], or photoinduced electron transfer [20]). Upon transformation of the quenching bioorthogonal unit in a specific ligation reaction, the fluorescence reinstates (Scheme 1). This two-in-one combination of bioorthogonal handles was exploited in the design of coumarin [21], BODIPY [16], phenoxazine [17], rhodamine [22], and cyanine-based [23], bioorthogonally applicable fluorogenic probes. It was observed, however, that the fluorescence increase upon bioorthogonal ligation, i.e., fluorogenicity, dramatically decreases towards the biologically preferred red range of the spectrum (Figure 1). To date, the most remarkable example of such fluorogenic probes in terms of fluorescence increase, are ultra violet (UV)/blue excitable ultrafluorogenic HELIOS (hyperemissive ligation-initiated orthogonal sensing) probes reported by Weissleder et al. in 2014 [21]. Emission of these coumarin-based probes was practically fully quenched by the bioorthogonal handle tetrazine. Upon specific reaction with *trans*-cyclooctenes (TCOs), 3–4 orders of magnitude increase in fluorescence were observed, which allowed no-wash fluorescence imaging of epidermal growth factor receptors (EGFR) using TCO labeled anti-EGFR antibodies.

To optimize probes for biological applications, it would be highly desirable to create fluorogenic dyes that are either excitable towards the red range or possess large Stokes-shift, while keeping the excellent fluorogenicity of UV/blue excitable systems. Our continuing efforts to address this problem resulted in the development of multiple fluorogenicity, as demonstrated on orange/far red excitable advanced probes [24,25]. Though these double fluorogenic probes solved the above-mentioned problems, their applicability is limited either to special sequences (i.e., double genetic incorporation of two non-canonical—cyclooctynylated—amino acids) [24] or microenvironments (i.e., the polar environment in the proximity of ligation) [25]. Thus, new approaches that could result in generally applicable, advanced probes are still needed. Since probes with large Stokes-shifts may also overcome the hampering effects of autofluorescence, we hypothesized that blue-green excitable, thus good fluorogenic probes having considerably large Stokes-shifts, may offer a more general solution to these problems.

Inspired by the outstanding fluorogenic performance of tetrazine-quenched coumarins, and the large Stokes-shifts of coumaryl–vinyl–pyridinium probes, we set forth a systematic study aimed at constructing small, π-extended fluorogenic probes on the grounds of HELIOS frames.

## 2. Materials and Methods

### 2.1. General

Unless otherwise indicated, all starting materials were obtained from commercial suppliers (Sigma-Aldrich (St. Louis, MO, USA), Merck (Darmstadt, Germany), Alfa Aesar (Haverhill, MA, USA), Reanal (Budapest, Hungary), Molar Chemicals (Halásztelek, Hungary), Fluorochem (Hadfield, Glossop, UK) and used without further purification. Analytical thin-layer chromatography (TLC) was performed on silica gel 60 F_254_ precoated aluminum TLC plates from Merck. Flash column chromatography was performed on Teledyne Isco CombiFlash^®^ Rf^+^ (Teledyne Isco, Lincoln, NE, USA) automated flash chromatography apparatus by using silica gel (25–40 μm) from Zeochem (Rüti, Switzerland). Microwave experiments were carried out on an AntonPaar (Graz, Austria) Monowave 300 microwave reactor (maximum power 850 W). NMR spectra were recorded on Varian Inova 500 MHz NMR spectrometer (Palo Alto, CA, USA). Chemical shifts (δ) are given in parts per million (ppm) by using solvent signals as the reference. Coupling constants (*J*) are reported in Hertz (Hz). Splitting patterns are designated as s (singlet), bs (broad singlet), d (doublet), dd (doublet of a doublet), t (triplet), q (quartet), or m (multiplet). Analytical reverse-phase high-performance liquid chromatography with ultra-violet/visible and mass spectrometry detection (RP-HPLC-UV/Vis-MS) experiments were performed on a SHIMADZU (Kyoto, Japan) LCMS-2020 system by using a Gemini C18 column (100 × 2.00 mm I.D.) with 5 mm silica (110 Å pore size) as a stationary phase with a photodiode array UV/Vis (λ = 220–800 nm) and an ESI-MS detector. Linear gradient elution (0 min 0% B; 2.0 min 100% B; 3.5 min 100% B; 4.5 min 0% B; 5.0 min 0% B) with eluents A (2% NH_4_HCO_3_, 5% MeCN, and 93% H_2_O) and B (2% NH_4_HCO_3_, 80% MeCN, and 18% H_2_O) was used at a flow rate of 1.0 mL min^−1^ at 30 °C. The samples were dissolved in a MeCN-H_2_O mixture. High resolution mass spectrometric measurements were performed using a Q-TOF Premier mass spectrometer (Waters Corporation, Milford, MA, USA) in positive electrospray ionization mode. Semipreparative HPLC was performed on a Hanbon (Huai’an, China) Semiprep NP7010C system using a Gemini C18 column (150 × 21 mm I.D.) with 5 μm silica (110 Å pore size) as a stationary phase.

### 2.2. Synthesis and Characterization

*7-(Diethylamino)-2-oxo-2H-chromen-4-yl trifluoromethanesulfonate* (**2**) [26], a mixture of compound 1 (6.0 g, 26 mmol, 1.00 equiv.), PhNTf_2_ (9.65 g, 27 mmol, 1.05 equiv.) and triethylamine (7.8 g, 10.8 mL, 77 mmol, 3.00 equiv.) was refluxed in dichloromethane (DCM) (100 mL) for 2 h. The reaction was cooled to room temperature, the solvent was removed in vacuo and the product was purified by flash column chromatography on silica gel (0–10 min 0–20% ethyl acetate in hexane) to give 6.8 g (72%) of **2** as a yellow solid. ^1^H NMR (500 MHz, CDCl_3_) δ 7.41 (d, *J* = 9.1 Hz, 1H), 6.64 (dd, *J* = 9.1, 2.5 Hz, 1H), 6.51 (d, *J* = 2.5 Hz, 1H), 6.04 (s, 1H), 3.43 (q, *J* = 7.1 Hz, 4H), 1.22 (t, *J* = 7.1 Hz, 6H). ^13^C NMR (126 MHz, CDCl_3_) δ 161.2, 158.1, 156.3, 152.1, 123.5, 118.5 (q, *J* = 320.8 Hz), 109.4, 102.0, 98.5, 97.4, 44.9, 12.3. MS: *m*/*z* calcd. for [C_13_H_15_NO_3_]^+^: 366, found: 366 [M + H]^+^.

*7-(Diethylamino)-4-(4-(6-methyl-1,2,4,5-tetrazin-3-yl)phenyl)-2H-chromen-2-one* (**5**), a mixture of compound 2 (98 mg, 0.27 mmol, 1.0 equiv.), 3-methyl-6-(4-(4,4,5,5-tetramethyl-1,3,2-dioxaborolan-2-yl)phenyl)-1,2,4,5-tetrazine [17] (80 mg, 0.27 mmol, 1.0 equiv.), PdCl_2_(dppf) (10 mg, 0.014 mmol, 0.05 equiv.) and CsF (82 mg, 0.54 mmol, 2.0 equiv.) was suspended in 3.0 mL of 1,4-dioxane and 0.3 mL of water. The reaction mixture was stirred at 80 °C for 20 min, cooled, diluted with DCM and extracted with water. The crude product was purified by flash column chromatography on silica gel (0–5 min 0–30% ethyl acetate in hexane) to give 48 mg (46%) of 5 as a red solid. For fluorescence measurements, the product was further purified with semipreparative HPLC (eluent: CH_3_CN, H_2_O). ^1^H NMR (500 MHz, CDCl_3_) δ 8.71 (d, *J* = 8.3 Hz, 2H), 7.66 (d, *J* = 8.3 Hz, 2H), 7.25 (d, *J* = 8.7 Hz, 1H), 6.60 (d, *J* = 2.5 Hz, 1H), 6.55 (dd, *J* = 8.9, 2.4 Hz, 1H), 6.07 (s, 1H), 3.42 (q, *J* = 7.1 Hz, 4H), 3.12 (s, 3H), 1.21 (t, *J* = 7.1 Hz, 6H). ^13^C NMR (126 MHz, CDCl_3_) δ 167.5, 163.7, 161.7, 156.8, 154.9, 150.6, 140.4, 132.6, 129.3, 128.2, 127.6, 108.9, 108.6, 98.2, 44.9, 21.2, 12.4. HRMS: *m*/*z* calcd. for [C_22_H_22_N_5_O_2_]^+^: 388.1774, found: 388.1770 [M + H]^+^.

*(E)-7-(Diethylamino)-4-(2-(6-methyl-1,2,4,5-tetrazin-3-yl)vinyl)-2H-chromen-2-one* (**6**), a mixture of compound **2** (25 mg, 0.0685 mmol, 1.0 equiv.), 2-(6-methyl-1,2,4,5-tetrazin-3-yl)ethyl methanesulfonate [27] (30 mg, 0.137 mmol, 2.0 equiv.), Pd_2_(dba)_3_ (3.1 mg, 0.0034 mmol, 0.05 equiv.), QPhos (4.9 mg, 0.0069 mmol, 0.1 equiv.) and dicyclohexylmethylamine (40 mg, 44 µL, 0.205 mmol, 3.0 equiv.) was dissolved in 1.00 mL of dry acetonitrile. The reaction mixture was stirred at 80 °C for 30 min, then cooled to room temperature and the volatiles were removed in vacuo. The crude product was purified by flash column chromatography on silica gel (0–5 min 0–30% ethyl acetate in hexane) to give 7 mg (30%) of **6** as a red solid. For fluorescence measurements, the product was further purified with semipreparative HPLC (eluent: CH_3_CN, H_2_O). ^1^H NMR (500 MHz, CDCl_3_) δ 8.52 (d, *J* = 16.1 Hz, 1H), 7.62 (d, *J* = 9.4 Hz, 1H), 7.60 (d, *J* = 16.1 Hz, 2H), 6.72 (d, *J* = 8.8 Hz, 1H), 6.63 (s, 1H), 6.40 (s, 1H), 3.45 (q, *J* = 7.0 Hz, 4H), 3.11 (s, 3H), 1.24 (t, *J* = 6.9 Hz, 6H). ^13^C NMR (126 MHz, CDCl_3_) δ 167.1, 163.9, 161.9, 156.6, 151.0, 148.3, 133.8, 128.3, 125.4, 108.8, 107.0, 105.9, 98.1, 44.8, 21.3, 12.4. HRMS: *m*/*z* calcd. for [C_18_H_20_N_5_O_2_]^+^: 338.1617, found: 338.1615 [M + H]^+^.

*3-Bromo-7-(diethylamino)-4-(4-(6-methyl-1,2,4,5-tetrazin-3-yl)phenyl)-2H-chromen-2-one* (**7**), compound **6** (48 mg, 0.124 mmol, 1.0 equiv.) was dissolved in tetrahydrofuran (4 mL) and was cooled to 0 °C, while *N*-bromosuccinimide (22 mg, 0.124, 1.0 equiv.) was added. Then the reaction mixture was stirred at room temperature for 30 min, then the volatiles were removed in vacuo. The crude product was purified by flash column chromatography on silica gel (0–15 min 0–30% ethyl acetate in hexane) to furnish 54 mg (94%) of **7** as a red solid. ^1^H NMR (500 MHz, CDCl_3_) δ 8.76 (d, *J* = 8.5 Hz, 2H), 7.53 (d, *J* = 8.5 Hz, 2H), 6.86 (d, *J* = 9.1 Hz, 1H), 6.57 (d, *J* = 2.5 Hz, 1H), 6.48 (dd, *J* = 9.1, 2.6 Hz, 1H), 3.41 (q, *J* = 7.1 Hz, 4H), 3.14 (s, 3H), 1.21 (t, *J* = 7.1 Hz, 6H). ^13^C NMR (126 MHz, CDCl_3_) δ 167.7, 163.9, 158.4, 155.4, 154.2, 151.1, 140.4, 132.6, 129.5, 128.4, 128.3, 109.3, 109.2, 104.3, 97.5, 45.0, 21.4, 12.6. HRMS: *m*/*z* calcd. for [C_22_H_21_BrN_5_O_2_]^+^: 466.0873, found: 466.0874 [M + H]^+^.

(E)-4-(2-(7-(Diethylamino)-4-(4-(6-methyl-1,2,4,5-tetrazin-3-yl)phenyl)-2-oxo-2H-chromen-3-yl)vinyl)-1-methylpyridin-1-ium iodide (**8**). In a microwave pressure tube with a magnetic stir bar compound **7** (9 mg, 0.019 mmol, 1 equiv.), 4-vinylpiridine (3 µL, 0.029 mmol, 1.5 equiv.), Pd_2_(dba)_3_ (1.8 mg, 0.0019 mmol, 0.1 equiv.), QPhos (1.4 mg, 0.0019 mmol, 0.1 equiv.), and dicyclohexylmethylamine (12,4 µL, 0.0579 mmol, 3.0 equiv.) was dissolved in 1.00 mL of absolute N,N-dimethylformamide. The mixture was heated in a microwave reactor at 80 °C for 60 min. The solvent was evaporated in vacuo and the crude products were purified by silica flash chromatography (0–15 min 0–10% methanol in DCM). The resulting intermediate was reacted with methyl iodide (12 µL, 0.193 mmol, 10 equiv.) in acetonitrile at 70 °C for 3 h. Then the volatiles were removed in vacuo and the crude products were purified by silica flash chromatography (0–15 min 0–70% ethyl acetate in hexane) to furnish 3.5 mg (27%) of **8** as a red solid. ^1^H NMR (500 MHz, CDCl_3_) δ 8.87 (d, *J* = 8.4 Hz, 2H), 8.79 (d, *J* = 6.6 Hz, 2H), 8.02 (d, *J* = 15.7 Hz, 1H), 7.55 (d, *J* = 8.3 Hz, 2H), 7.52 (d, *J* = 6.8 Hz, 2H), 7.15 (d, *J* = 15.8 Hz, 1H), 6.92 (d, *J* = 9.1 Hz, 1H), 6.58 (d, *J* = 2.5 Hz, 1H), 6.54 (dd, *J* = 9.2, 2.6 Hz, 1H), 4.50 (s, 3H), 3.47 (q, *J* = 7.1 Hz, 4H), 3.17 (s, 3H), 1.25 (t, *J* = 7.1 Hz, 6H). ^13^C NMR (126 MHz, CDCl_3_) δ 167.9, 163.6, 160.0, 157.1, 156.4, 154.9, 152.5, 144.4, 138.2, 135.8, 133.5, 130.1, 129.9, 128.7, 124.6, 123.4, 112.2, 110.1, 109.5, 97.2, 48.4, 45.4, 21.4, 12.7. HRMS: *m*/*z* calcd. for [C_30_H_29_N_6_O_2_]^+^: 505.2346, found: 505.2344 [M]^+^.

### 2.3. Spectroscopic Measurements

Stock solutions of dyes were prepared in 1 mM concentration in DMSO. Reactions with (1*R*,8*S*,9s)-Bicyclo[6.1.0]non-4-yn-9-ylmethanol (BCN) were carried out in these stock solutions by adding an excess of solid BCN to the solution. The reaction was monitored by LC-MS until the reaction went to completion. Subsequent fluorescence measurements were carried out by diluting stock solutions to 5 µM concentrations with PBS. Spectroscopic measurements were performed on a Jasco FP 8300 spectrofluorometer (Halifax, NS, Canada) and a JASCO v750 spectrophotometer (Halifax, NS, Canada) in PBS (pH = 7.4) at room temperature (r.t.). The excitation and emission slits were set to 2.5 nm, scanning speed was set to 500 nm/min. All measurements were carried out in quartz cuvettes with a path length of 1 cm. Clear PBS (phosphate buffered saline) solution with additional DMSO was used for blank measurements. Each dye was excited at the respective excitation maxima and fluorescence was detected at the corresponding emission maxima. Turn-on values were calculated by dividing the start and end fluorescence intensity values of time-course measurements at the emission maxima of the products. Quantum yields were determined using coumarin 153 in ethanol (Φ = 0.55) [28] and rhodamine B in basic ethanol (Φ = 0.65) [29] as standard.

### 2.4. Cell Culture

COS-7 cells (Sigma 87021302, St. Louis, MO, USA) or HeLa (ATCC CCL-2) were maintained in Dulbecco’s modified Eagle’s medium (Life Technologies, ThermoFisher Scientific, Waltham, MA, USA, 41965-039) supplemented with 1% penicillin–streptomycin (Sigma P0781, St. Louis, MO, USA), 1% l-Glutamine (Sigma G7513, St. Louis, MO, USA), 1% sodium pyruvate (Life Technologies 11360, ThermoFisher Scientific, Waltham, MA, USA), and 10% FBS (Sigma F7524, St. Louis, MO, USA). Cells were cultured at 37 °C in a 5% CO_2_ atmosphere and passaged every 3–4 days up to 20 passages.

### 2.5. Live Cell Vimentin Labeling

COS-7 cells were transferred into ibidi μ-slide 8-well glass-bottom plates (15,000 cell/well) and incubated for 20–24 h at 37 °C in a 5% CO_2_ atmosphere. Cells were transfected with 0.25 μg Vimentin^N116TAG^-PSmOrange and tRNA^Pyl^/NES-PylRS^AF^ plasmids [24,30] (kind gift of Edward Lemke’s lab) with Jetprime transfection agent according to its protocol. Cells were incubated in 250 μM BCN-lysine (Sichem, Bremen, Germany, SC-8014) containing complete medium during the transfection and the following 24 h period. Afterward, the supernatant was replaced by non-canonical amino acid (ncAA) free medium for overnight. On the fourth day, cells were labeled with the fluorogenic dye **8** in 1 μM concentration (in complete DMEM, Dulbecco’s Modified Eagle Medium) for 60 min at 37 °C in the dark. After a washing step (2 h), cells were fixed (4% paraformaldehyde (PFA) for 10 min at 25 °C) and washed again with PBS twice and were imaged. In the case of a no-wash scheme, cells were treated with 0.1 μM concentration of **8** and were imaged without a washing step.

### 2.6. Dual Color Labeling

#### 2.6.1. Immunostaining

COS-7 cells were transferred into ibidi μ-slide 8-well glass-bottom plates (15,000 cell/well) and incubated for 20–24 h at 37 °C in a 5% CO_2_ atmosphere. Cells were washed three times with DPBS (PBS containing 0.9 mM CaCl_2_ and 0.5 mM MgCl_2_) and fixed and permeabilized with 3.8% PFA and 0.1% Triton X-100 for 5 min. After washing samples were blocked with complete blocking solution (2 mg/mL BSA, 1% fish gelatin (Sigma G7765, St. Louis, MO, USA) and 15 TritonX-100 in DPBS) for an hour at room temperature. The primary antibody (rabbit anti-TOMM20, Abcam (Cambridge, UK) ab186734) was incubated in 1:250 dilution for an hour. After washing, the samples were treated with the secondary antibody fluorescently modified by dye **8** for 60 min in the dark.

#### 2.6.2. Actin Labeling

Sequential actin labeling was carried out with Cy3 [23] modified Phalloidin in TBS (tris-buffered saline) buffer for 40 min in the dark. Plates were washed and imaged.

### 2.7. Confocal and STED Imaging and Analysis

Confocal and STED images were acquired on a Leica TCS SP8 STED 3X microscope using the 488 or 552 nm laser for excitation; 660 nm STED (1.5 W, continuous wave) laser for depletion. The images were taken using a Leica HC PL APO 100×/1.40 oil immersion objective along with a Leica HyD detector. Using the Huygens STED Deconvolution Wizard (Huygens Software, Hilversum, The Netherlands), only a moderate degree of deconvolution was applied to the recorded STED images to avoid deconvolution artifacts. The deconvolution was based on theoretical point spread function (PSF) values. Images were analyzed using Leica (Weitzlar, Germany) Application Suite X and ImageJ software (NIH). Gaussian non-linear fitting and full-width at half maximum values (FWHM) were obtained with Origin Pro 9 software (Northampton, MA, USA).

## 3. Results and Discussion

Considering the possible means for the extension of the π-system of coumarins with a vinylpyridynium motif, either the 3rd or the 4th position seems an obvious choice for synthetic reasons. Literature examples show, however, that 4-(vinylpyridyl)coumarins lose their fluorescence [31], leaving 3-(vinylpyridyl)coumarins as possible π-extended frames. Such 3-(vinylpyridyl)coumarin probes possess remarkable Stokes-shifts (often referred to as megaStokes dyes, [32]) and red-shifted (ca. 80–100 nm) excitation and emission maxima with respect to plain coumarin [32,33]. However, direct modification of the original HELIOS frame is rather limited and does not allow ready extension of the π-system, for the 3rd position is already taken by the quenching phenyl–methyl tetrazine moiety. Thus, we first needed to examine whether a similar quenching effect of phenyl tethered tetrazines is exerted at the 4th position. Moreover, we also intended to study the quenching effect of a vinylene tethered tetrazine. To this end, we first explored the possibility of installing the tetrazine unit onto the 4th position of the coumarin scaffold via a vinylene or phenylene linker. Recently, we demonstrated the suitability of Suzuki-type of cross-coupling reactions for the direct installation of phenylene linked tetrazines onto various fluorescent cores [17,23]. Furthermore, Heck cross-coupling of in situ generated vinyltetrazines, a method developed by the Devaraj group, is also a feasible means for the introduction of the tetrazine motif [27].

**Scheme 2:**
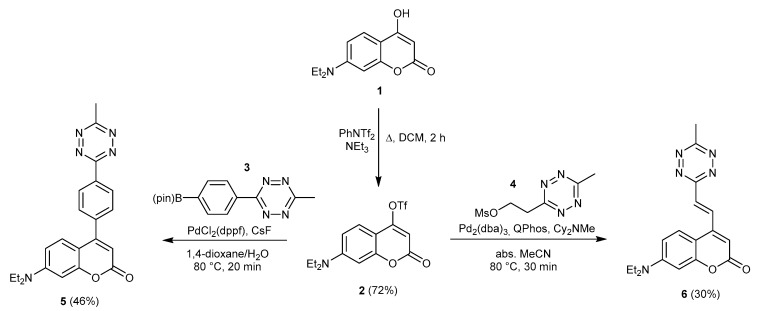
Synthesis of hyperemissive ligation-initiated orthogonal sensing (HELIOS)-like probes **5** and **6.**

To access such 4- substituted coumarins, we synthesized 7-(diethylamino)-2-oxo-2*H*-chromen-4-yl trifluoromethanesulfonate (**2**) and treated it either with boronate **3** or mesylate **4** in the presence of a Pd catalyst, furnishing the respective 4-functionalized coumarins (**5** and **6**) in medium yields (Scheme 2).

With these HELIOS-like probes in hand, we tested their fluorogenic performance by comparing the fluorescence spectra of the parent tetrazines and their reaction products with cyclooctyne, **BCN** ((1*R*,8*S*,9s)-Bicyclo[6.1.0]non-4-yn-9-ylmethanol) (Scheme 1, Table 1, and Figure S1). We found that both **5** and **6** were practically fully quenched. **BCN** conjugate of **5**, on the other hand, showed intense fluorescence. At the same time, **6-BCN** was found to be poorly fluorescent. For both probes, we observed practically fully quenched spectra. Therefore, the determination of fluorescence enhancement values, FE (FE = I/I_0_, where I is the fluorescence intensity at λ_em_ of the respective **BCN** conjugate, and I_0_ is the intensity of the unconjugated probe at the λ_em_ of the **BCN** conjugate) was meaningless. Thus, we instead called this fluorogenicity infinite.

**Scheme 3:**
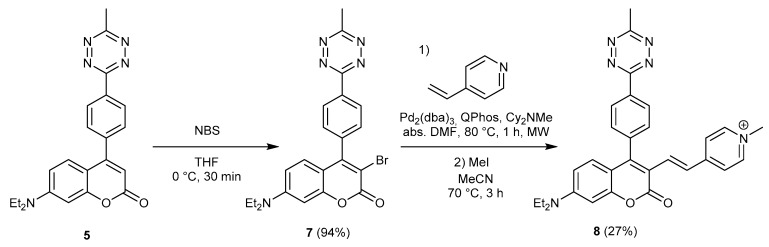
Synthesis of π-extended HELIOS-like probe **8**.

Knowing that 4-tethered phenylene-tetrazines also exert full quenching of the coumarin fluorescence, which can be reinstated upon reaction with a strained alkyne, we aimed at extending the π-conjugation of the fully quenched **5** scaffold by installing a 1-methyl-4-vinylpyridinyl at the 3rd position, to access red-shifted HELIOS-like fluorogenic probe. We proposed a synthetic routine, which utilizes Heck-cross coupling for the incorporation of the vinyl-pyridyl motif. To this end, **5** was treated with *N*-bromosuccinimide (NBS) to access 3-bromo functionalized intermediate **7**, which was subsequently reacted with 4-vinylpyridine in the presence of a Pd catalyst. The resulting π-extended coumarin was further treated with MeI to obtain a red-shifted Helios-like probe **8** in medium yield (Scheme 3).

Following synthesis, we have explored the spectral characteristics of probe **8** and its in situ formed **BCN** conjugate. To our delight, the excitation maximum in PBS buffer was found to be at around 488 nm, perfectly matching with one of the most frequently used microscope laser-line, while emission maximum was centered at 600 nm, in accordance with the large Stokes shift of related compounds [32,33]. Gratifyingly, a comparison of the fluorescence intensities of **8** and **8-BCN** revealed a ca. 30-fold increase in quantum yield at 600 nm (for spectra see Figure S1). We did not observe any considerable changes in emission intensities between pH 4–9 either for **8** or **8-BCN** (Figure S3).

Next, we aimed at demonstrating the applicability of our new, fluorogenic large Stokes-shift probe in protein labeling schemes. First, we applied the probe to our fixed-cell labeling model. COS-7 cells were fixed and treated with a cyclooctynylated actin affinity tag, **BCN**-phalloidin, then washed. Subsequently, the tagged actin filaments were stained with probe **8**, washed, and subjected to confocal microscopy. This experiment was repeated without the washing step to explore the fluorogenic nature of the probe (Supplementary Material, Figure S4). There was no substantial difference between the two image sets in terms of background fluorescence indicating that the extent of fluorescence increase is enough to differentiate between specific and non-specific staining. It is worth mentioning that labeling was efficient even at 0.1 μM labeling concentration. Next, we aimed at testing the probe in a more challenging environment, i.e., in live-cell labeling schemes. First, we assessed the toxicity of probe **8** on COS-7 cells. The presence of the probe did not affect the viability of the cells within a reasonable concentration range (0–30 μM) used for labeling experiments, as indicated by the 3-(4,5-dimethylthiazol-2-yl)-2,5-diphenyltetrazolium bromide (MTT) assay (Figure S6). To test probe **8** on live cells, a tailored, genetically bioorthogonalized cytoskeletal protein, vimentin, modified with a non-canonical, bioorthogonalized lysine (Lys(^ε^N-**BCN**_endo_) was expressed in COS-7 cells, using the orthogonal pyrrolysine tRNA^Pyl^/PylRS pair from *Methanosarcina mazei*. The construct also carried a *C*-terminal mOrange fusion tag for reference (Vim^N116TAG^-mOrange).

**Figure 2:**
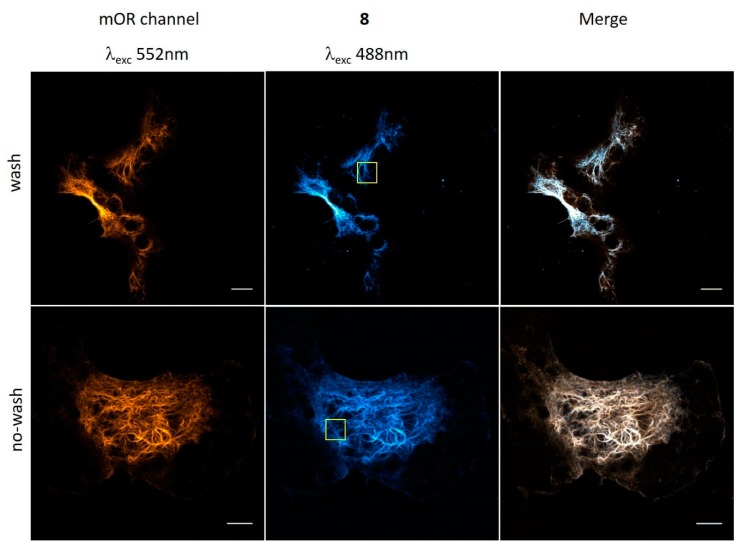
Confocal microscopy images of Vim^Lys-**BCN**^-mOrange expressing COS-7 cells labeled with **8** (washed and no-washed schemes). Scale bar: 10 µm. Yellow squares indicate the magnified area presented in Figure 3.

Live COS-7 cells were then treated with probe **8**, washed, fixed, and subjected to confocal microscopy. Images of the as-treated COS-7 cells showed good co-localization of the probe with the reference mOrange, suggesting specific labeling at the position of the Lys-**BCN** (Figure 2). This experiment also indicated that probe **8** is membrane permeable and can stand the conditions of live cell labeling. We were curious whether the fluorogenic nature of **8** allows no-wash labeling schemes in live-cell labeling and repeated the labeling process without the washing and fixation steps. To our delight, images of live cells showed specific, low background labeling of vimentin filaments (Figure 2).

**Figure 3:**
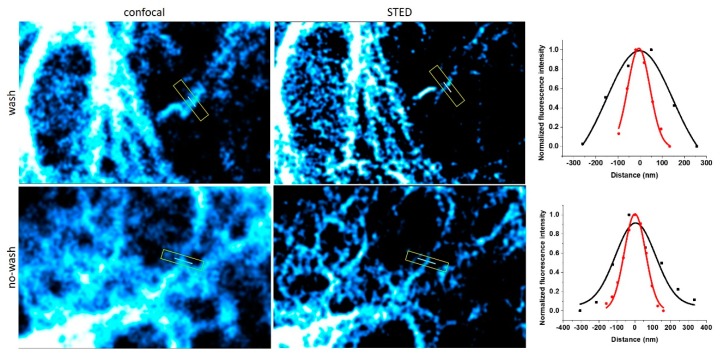
Confocal and STED microscopy images of Vim^Lys-**BCN**^-mOrange expressing COS-7 cells labeled with **8** (λ_exc_: 488 nm, CW 660 nm depletion laser). Full-width at half maximum (FWHM) values for confocal (black) and STED images (red) were 351 ± 95 and 118 ± 10 nm for washed and 264 ± 60 and 140 ± 8 nm for no-washed samples, respectively.

The spectral properties of **8** suggest that the probe is suitable for super-resolution imaging using STED microscopy with a 660 nm depletion laser (Supplementary Material, Figure S2). Therefore, we have subjected the samples to super-resolution imaging using STED microscopy (Figure 3) and concluded that probe **8** is indeed suitable for STED microscopy. Again, we did not experience considerable change in the resolution between washed and non-washed samples. It should also be noted that no loss of signal-to-noise ratio was observed up to four cycles of STED imaging with ~64% STED intensity. Further cycles, however, resulted in gradually fading images due to bleaching of the probe (Figure S5).

**Figure 4:**
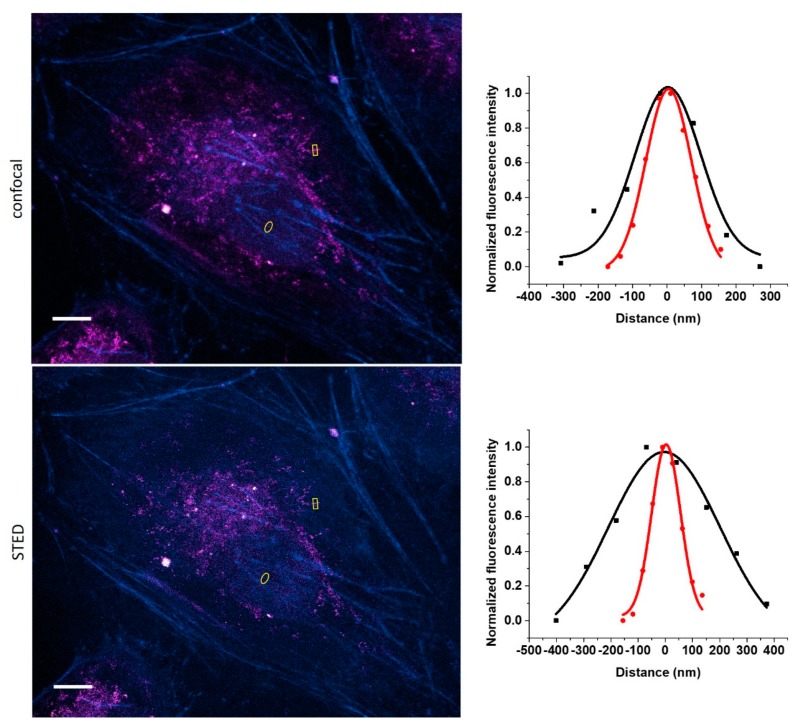
Confocal and STED microscopy images of IgG-**8** labeled mitochondrial protein TOMM20 (magenta) and Cy3-phalloidin labeled actin filaments (cyan). Images were excited at 488 nm (**8**) and 552 nm (**Cy3**), while detected with the same detection window (580–650 nm). For STED images, a CW 660 nm depletion laser was used. Full-width at half maximum (FWHM) values for confocal (black) and STED images (red) were 224 ± 46 and 155 ± 10 nm for TOMM20 (upper-right) and 483 ± 76 and 124 ± 8 nm for actin (lower-right), respectively. Scale bar: 10 µm.

Besides reducing autofluorescence, probes having large Stokes-shifts are also quite useful in terms of addressing a common problem present in multicolor labeling schemes and imaging. Lenses tend to fail to focus equally on all colors to the same points due to dispersion. This phenomenon, chromatic aberration [34], thus often compromises super-resolved images where more colors are used. Dyes that are excitable at distinct wavelengths but having similar emission windows can overcome this problem. Probe **8** seems ideal from this respect, as, due to its large Stokes-shift, it can be paired with other probes, having smaller Stokes-shift and emitting at around 600 nm. To demonstrate this, we applied a dual-color labeling scheme involving mitochondrial protein TOMM20 and actin filaments. Fixed HeLa cells were treated with anti-TOMM20 IgG and a secondary antibody tagged with **8**, while actin filaments were stained with Cy3 [23] stained affinity tag, phalloidin. The two probes, **8** and Cy3, were excited at 488 nm and 552 nm, respectively, while detection was carried out at the same emission window. Images were also subjected to STED microscopy, giving rise to dual-color STED imaging (Figure 4).

## 4. Conclusions

Herein, we presented the synthesis and application of a fluorogenic, large Stokes-shift, bioorthogonally applicable, membrane-permeable tetrazine probe. We studied the possibility of extending the π-electron system of ultra-fluorogenic HELIOS probes and concluded that the quencher and bioorthogonal motif tetrazine can exert similar quenching of emission when it is placed at the 4th position of the coumarin frame. This finding enabled us to extend the conjugation of the tetrazine–coumarin via the 3rd position, giving rise to probe **8**, excitable at common laser line 488 nm with emission range at around 600 nm. Cell labeling studies revealed that the probe is membrane permeable and can stand the challenging environment of live cells. Furthermore, the ca. 30-fold fluorogenicity allowed reduced background and consequently, no-wash imaging schemes, which is evidently advantageous from a practical perspective, but also, it might enable studies of proteins with rapid turnover times, such as many membrane receptors, nuclear proteins, or MAP kinases. The large Stokes-shift can also be exploited in multi-color labeling schemes and can address the problematics of chromatic aberration in combination with small Stokes-shift dyes emitting around 600 nm.

## Figures and Tables

**Table 1:** Main photophysical data of probes **5**, **6,** and **8** and their (1*R*,8*S*,9s)-Bicyclo[6.1.0]non-4-yn-9-ylmethanol (BCN) conjugates. ^1^

	λ_abs_ (nm)	λ_em_ (nm)	ε (M^−1^ cm^−1^)	φ	B (ε × φ)
**5**	386	n.d.	9800	n.d.	n.a.
**5-BCN**	400	542	13000	0.041 ^2^	533
**6**	452	n.d.	6700	n.d.	n.a.
**6-BCN**	429	n.d.	9800	n.d.	n.a.
**8**	495	620	24000	0.0021 ^3^	50.4
**8-BCN**	489	614	23000	0.057 ^3^	1311

^1^ in PBS. ^2^ Relative to coumarin 153 (Φ = 0.55) [28], ^3^ Relative to rhodamine B (Φ = 0.65) [29].

## Supplementary Materials

The following are available online at https://www.mdpi.com/2218-273X/10/3/397/s1, synthesis of starting materials, excitation, and emission spectra of probe **8-BCN**, NMR spectra of all compounds, details of actin labeling, fluorescent modification of secondary antibody, capillary electrophoresis, Figure S1: Emission spectra of probes **5**, **6,** and **8** and their **BCN** conjugates in PBS (λ_exc_= 488 nm), Figure S2: Spectral bands of **8-****BCN** in PBS with excitation (488 nm) and depletion (660 nm) laser lines, Figure S3: Emission intensities of **8** and **8-BCN** at maxima at different pH values, Figure S4: Confocal and STED microscopy images of actin in COS-7 cells labeled bioorthogonally with **BCN**-phalloidin and dye **8** (λ_exc_: 488 nm, CW 660 nm depletion laser), Figure S5: Photostability of dye **8**, Figure S6: The effect of dye **8** and Doxorubicin (Dox) on cell viability.
