# Supplementary material for: A Bioorthogonally Applicable, Fluorogenic, Large Stokes-Shift Probe for Intracellular Super-Resolution Imaging of Proteins
Source: Biomolecules. 2020 Mar 4;10(3):397. doi: 10.3390/biom10030397 (PMC7175155; doi:10.3390/biom10030397)
Supplement: Supplementary file 1 [file biomolecules-10-00397-s001.pdf]

Supplementary Material for

A Bioorthogonally Applicable, Fluorogenic, Large Stokes-Shift Probe for Intracellular Super-Resolution Imaging of Proteins

Evelin Németh, Gergely Knorr, Krisztina Németh and Péter Kele*

Chemical Biology Research Group, Institute of Organic Chemistry, Research Centre for Natural Sciences. H-1117 Magyar tudósok krt. 2, Budapest, Hungary

CONTACT

Péter Kele: kele.peter@ttk.hu

Syntheses and characterization

3-(4-bromophenyl)-6-methyl-1,2,4,5-tetrazine (S1) [1-2]

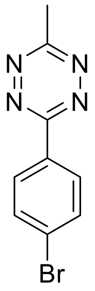

3-(4-bromophenyl)-6-methyl-1,2,4,5-tetrazine was synthesized according to the literature, with modifications.

A mixture of 4-bromobenzonitrile (1.00 g, 5.5 mmol, 1.0 equiv.) and $Zn(OTf)_2$ (1.00 g, 2.75 mmol, 0.5 equiv.) was heated at 100 °C in hydrazine monohydrate (10 mL) for 30 minutes. The reaction mixture was cooled to 50 °C and acetamidine hydrochloride (10 g) was added over 1 h, portion-wise. After the addition of acetamidine hydrochloride, water (10 mL), ethyl acetate (10 mL), and $NaNO_2$ (10 g), the reaction mixture was cooled to 0 °C. Concentrated HCl was slowly added dropwise until the bubbling of NO_x stopped, and the pH of the water phase was acidic. The water phase was washed with ethyl acetate multiple times. The combined organic layers were dried over $MgSO_4$, and the solvent was removed in vacuo. The crude product was purified by flash column chromatography on silica gel (0–5 min 0%–10% ethyl acetate in hexane) to give 0.76 g (55%) of red solid. ^{1}H NMR (500 MHz, $CDCl_3$) δ 8.42 (d, J = 7.9 Hz, 2H), 7.69 (d, J = 7.8 Hz, 2H), 3.07 (s, 3H). ^{13}C NMR (126 MHz, $CDCl_3$) δ 167.5, 163.6, 132.6, 130.8, 129.4, 127.8, 21.3. MS: m/z calcd. for $[C_9H_7BrN_4]^+$: 251, found: 251 $[M+H]^+$.

3-methyl-6-(4-(4,4,5,5-tetramethyl-1,3,2-dioxaborolan-2-yl)phenyl)-1,2,4,5-tetrazine (3) [1]

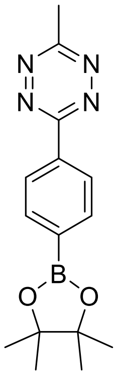

The synthesis of compound **3** was based on the literature. A mixture of compound **S1** (500 mg, 2.00 mmol, 1.0 equiv.), $B_2(pin)_2$ (557 mg, 2.2 mmol, 1.1 equiv.), $PdCl_2(dppf)$ (73 mg, 0.1 mmol, 0.05 equiv.), and KOAc (293 mg, 3.0 mmol, 1.5 equiv.) was refluxed in 1,4-dioxane (10 mL) for 2 h. After 2 h, the solution was cooled to room temperature and concentrated in vacuo. The residue was dissolved in CH_2Cl_2 (20 mL) and washed with water. The organic layer was dried over anhydrous $MgSO_4$, filtered, and concentrated in vacuo. The crude product was purified by flash chromatography on silica gel (0–5 min 0%–30% DCM in hexane) to give 0.32 g (54%) of red solid. ^{1}H NMR (500 MHz, $CDCl_3$) δ 8.53 (d, J = 8.2 Hz, 2H), 7.98 (d, J = 8.2 Hz, 2H), 3.05 (s, 3H), 1.35 (s, 12H). ^{13}C NMR (126 MHz, $CDCl_3$) δ 167.3, 164.2, 135.5, 134.1, 127.0, 84.3, 25.0, 21.2. MS: m/z calcd. for $[C_{15}H_{19}BN_4O_2]^+$: 299, found: 299 $[M+H]^+$.

7-(diethylamino)-4-hydroxy-2H-chromen-2-one (1) [3]

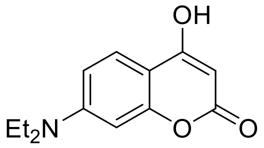

The synthesis of compound **1** was based on the literature. A mixture of 3-diethylamino phenol (11.6 g, 70 mmol, 1.0 equiv.) and diphenyl malonate (18.0 g, 70 mmol, 1.0 equiv.) was refluxed in toluene (120 mL) overnight. The mixture was cooled to room temperature, filtered, washed with toluene and hexane, and dried to give 8.55 g (52%) of gray powder. ^{1}H NMR (500 MHz, DMSO) δ 11.86 (s, 1H), 7.54 (d, J = 9.0 Hz, 1H), 6.66 (dd, J = 9.0, 2.5 Hz, 1H), 6.45 (d, J = 2.4 Hz, 1H), 5.25 (s, 1H), 3.41 (q, J = 7.0 Hz, 4H), 1.12 (t, J = 7.0 Hz, 6H). ^{13}C NMR (126 MHz, DMSO) δ 166.4, 162.7, 156.1, 150.8, 124.1, 108.1, 103.4, 96.4, 86.1, 43.9, 12.3. MS: m/z calcd. for $[C_{13}H_{15}NO_3]^+$: 234, found: 234 $[M+H]^+$.

Spectral measurements

Emission spectra

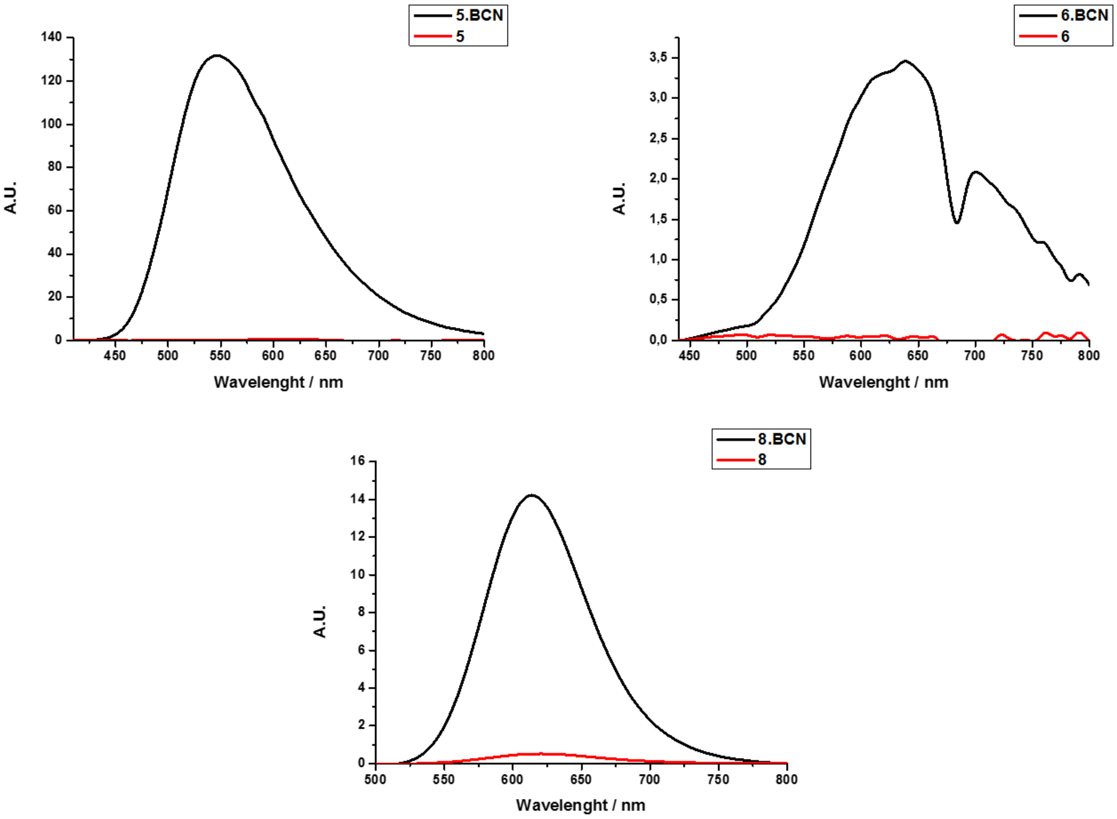

Figure S1. Emission spectra of probe **5**, **6**, **8** and their **BCN** conjugates in PBS (λ_{exc}= 488 nm)

Excitation and emission spectra of probe 8.BCN

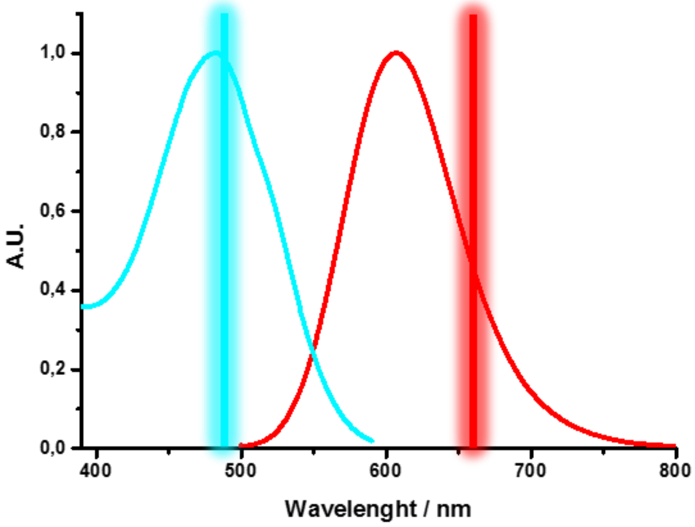

Figure S2. Spectral bands of **8-BCN** in PBS with excitation (488 nm) and depletion (660 nm) laser lines.

pH-dependent emission of probe 8

The fluorescence of compound **8** and its **BCN** conjugate measured in phosphate buffer at different pH values.

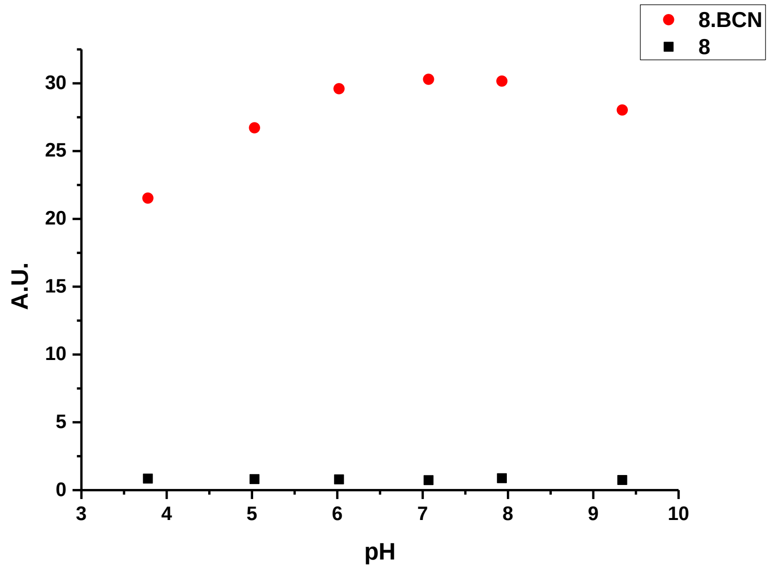

Figure S3. Emission intensities of **8** and **8-BCN** at maxima at different pH values.

Actin labeling [4]

Actin labeling was performed based on the procedure published by Wieczorek et al. [5] and Meimetis et al. [6]. Synthesis of phalloidin-**BCN** was performed per Egyed et al. [7]. COS-7 cells were transferred into ibidi µ-slide 8-well glass-bottom plates (15,000 cell/well) and incubated for 20–24 h at 37 °C in a 5% CO_2 atmosphere. Cells were washed with PBS, then fixed (4% PFA for 10 min at 25 °C) and permeabilized (0.1% Triton X-100 for 5 min at 25 °C). Phalloidin-**BCN** treatment was carried out in a final concentration of 1 µg/mL (200 µg/mL stock concentration in MeOH), and freshly diluted in labeling buffer (10 mM TBS (pH 7.4), 0.1% Triton X-100 and 2% BSA) for 40 min at 25 °C. After the washing step, cells were labeled with the fluorogenic dye **8** in 1 µM concentration (in PBS) for 60 min at 25 °C in the dark). Cells were washed with PBS twice and were imaged. In the case of the no-wash scheme, cells were treated with 0.1 µM concentration of **8** and were imaged without a washing step.

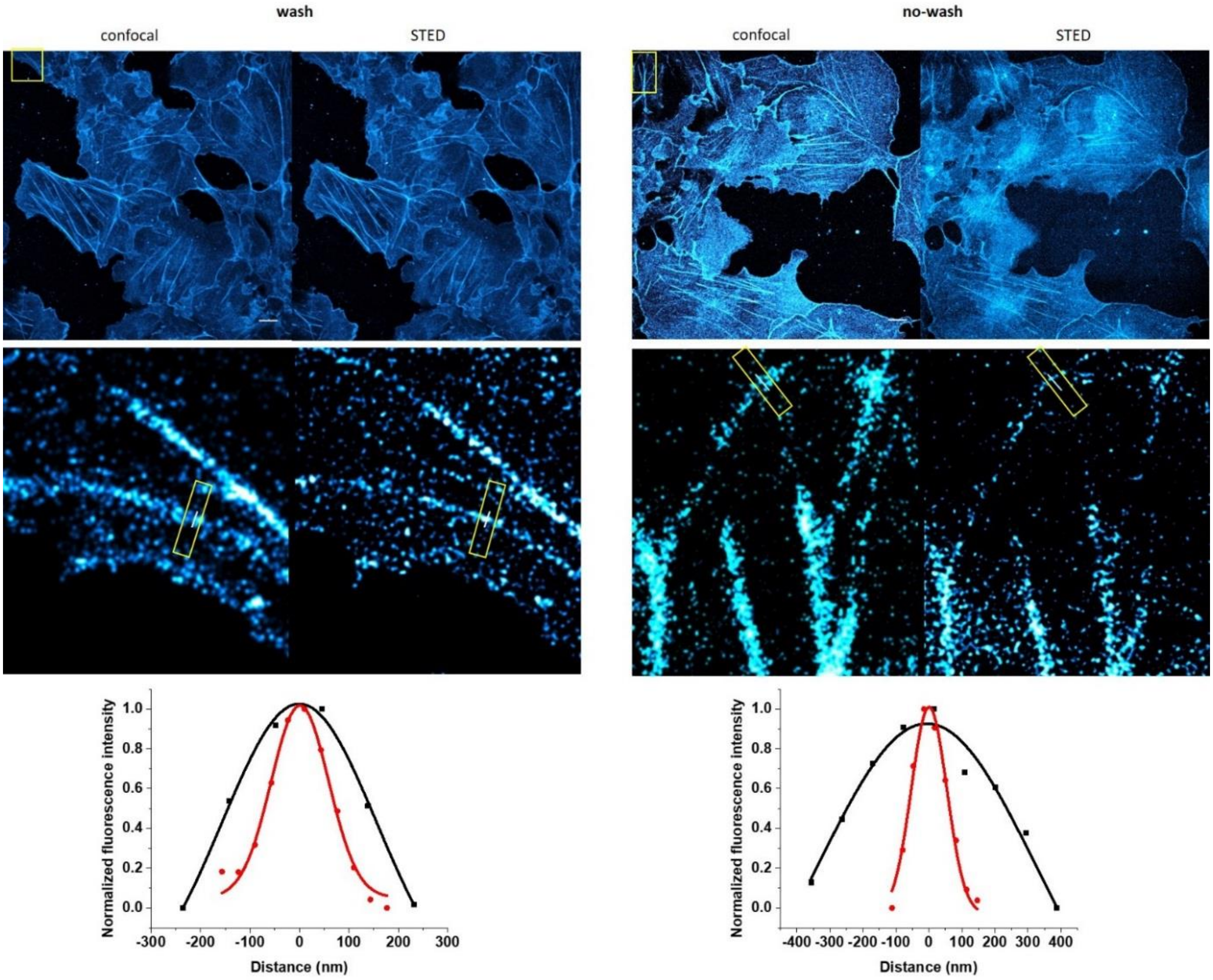

Figure S4. Confocal and STED microscopy images of actin in COS-7 cells labeled bioorthogonally with BCN-phalloidin and dye **8** (λ_{exc}: 488 nm, CW 660 nm depletion laser). Full-width at half maximum (FWHM) values for confocal (black) and STED images (red) were 368±56 and 137±10 nm for washed and 777±359 and 126±10 nm for non-washed samples, respectively. Scale bar 10 µm.

Dual-color labeling

Fluorescent modification of secondary antibody. As a first step buffer, exchange (to 110 mM $NaHCO_3$/ Na_2CO_3, pH 9.0) of goat anti-rabbit IgG samples (Jackson ImmunoResearch, 111-005-003) was carried out using Sephadex G25 “Fine” desalting gel (Pharmacia Fine Chemicals, Sweden) with SpinPrep column technology (Sigma, St Louis, MO, USA). (1*R*,8*S*,9s)-bicyclo[6.1.0]non-4-yn-9-ylmethyl *N*-succinimidyl ester (NHS-**BCN**; Sigma–Aldrich, cat no.: 744867) was added to the IgG in 50 µM concentration at room temperature for 20 mins. Afterward, in a one-pot reaction, dye **8** was added at 112 µM concentration for an additional 20 mins. The excess reagents were removed by using the desalting G25 spin column described above. Reaction yields, IgG concentration, and purity were checked by the capillary electrophoresis method.

Capillary electrophoresis. Background electrolyte (BGE) components boric acid was purchased from Sigma (St. Louis, MO, USA) sodium hydroxide, sodium carbonate, and bicarbonate were purchased from Merck GmbH (Darmstadt, Germany).

Capillary electrophoresis was performed with an Agilent Capillary Electrophoresis $3D^{CE}$ system (Agilent Technologies, Waldbronn, Germany) applying bare fused capillary having a 64.5 cm total and 56 cm effective length with 50 µm I.D. (Agilent Technologies, Santa Clara, CA, USA). On-line absorption at 200 nm was monitored for the protein and 488 nm for dye **8** by a DAD UV-Vis detector. A laser (λ_{ex}: 488 nm) (Melles Griot, Carlsbad, USA) induced fluorescent (LIF) signal was observed by a ZetaLIF Discovery detector (Picometrics, Labege, France) at 45.5 cm effective length. The capillary was thermostated at 25 °C. Between measurements, the capillary was rinsed subsequently with 0.1 M HCl, 1.0 M NaOH, 0.1 M NaOH, and distilled water for 3 minutes each and with BGE (200 mM sodium borate (pH 9.0)) for 5 minutes. Samples were injected by 5×10^3 Pa pressure for 6 sec. Runs were performed in the positive-polarity mode with 30 kV.

Synthesis of Phalloidin-Cy3

Aminophalloidin (Bachem H7634.0001; 60 µg, 70 nmol, 1.0 equiv. – *70 µL from the 1 mM stock solution*), **BCN**-NHS (Sigma 744867; 377 µg, 1.1 µmol, 15.0 equiv. – *11 µL from the 100 mM stock solution*) and Cy3-tetrazine [4], 940 µg, 1.6 µmol, 22 equiv. – *16 µL from the 100 mM stock solution*) was mixed in 110 mM Na_2CO_3/$NaHCO_3$ + 2.2 mM EDTA buffer (620 µl). The reaction mixture was incubated in the dark at 25 °C for 2 h, then purified with reversed-phase HPLC on a Phenomenex Jupiter 10 µm C18 250 mm x 10 mm column (H_2O:MeCN both with 0.1% HCOOH, gradient elution from 5% to 75% MeCN in 30 min) to give phalloidin-Cy3.

Photostability of dye 8 in STED conditions

No loss of signal-to-noise ratio could be detected for 4 cycles of STED imaging using 64% laser intensity, as we demonstrated in Figure S5. Further cycles, however, resulted in faded images due to bleaching of the fluorescent dye. For the experiment, COS-7 cells were fixed and treated with **BCN**-phalloidin and stained with probe **8** (1 µM) in no-wash condition and subjected to confocal microscopy.

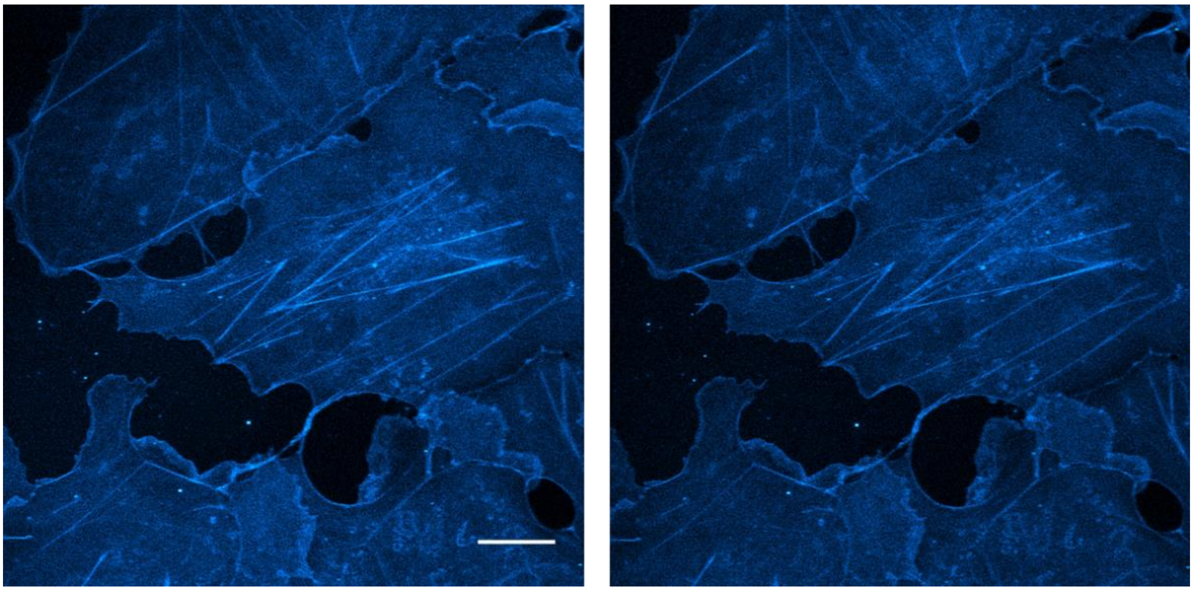

1st cycle **4th cycle**

Figure S5. Photostability of dye **8**. STED microscopy images of actin in COS-7 cells labeled bioorthogonally with **BCN**-phalloidin and dye **8** under no-wash condition. No loss of signal to noise ratio was detected until the 4th cycle compared to the first (left) and the fourth (right) cycle STED image **8** (λ_{exc}= 488 nm, CW 660 nm depletion laser; 64% STED intensity). Scale-bar:10 µm.

Effect of dye 8 on cells

An MTT test was carried out for the investigation of cytotoxicity of dye **8**.

COS-7 cells were transferred into a 48-well plate (Greiner-Bio-One) (15,000 cell/well) and incubated for 20–24 h at 37 °C in a 5% CO_2 atmosphere. Cells were treated with 0.1–30 µM of **8** for 3 or 24 hours. Doxorubicin (Dox) in the concentration range of 0.1–10 µM for 24 h was applied as a positive control. After the incubation period, supernatants were replaced with 0.5 mg/ml MTT (3-(4,5-dimethylthiazol-2-yl)-2,5-diphenyltetrazolium bromide) solution (in complete DMEM) and incubated for 150 min at 37 °C in the dark. The insoluble formazan crystals were dissolved in DMSO. Absorbance was detected at 540 nm using a Biotek Synergy 2 Cytation 3 imaging plate reader with Gen5 software version 3.08 (Biotek Winooski, VT, USA).

The estimated IC_{50} value for Dox was 163 nM. At the same time, no significant difference (calculated by paired *t*-test OriginPro software ver. 9) could be detected in the viability of the COS-7 cells treated with **8**— up to 25 µM (incubated for 24 hours)— compared to the untreated cell population.

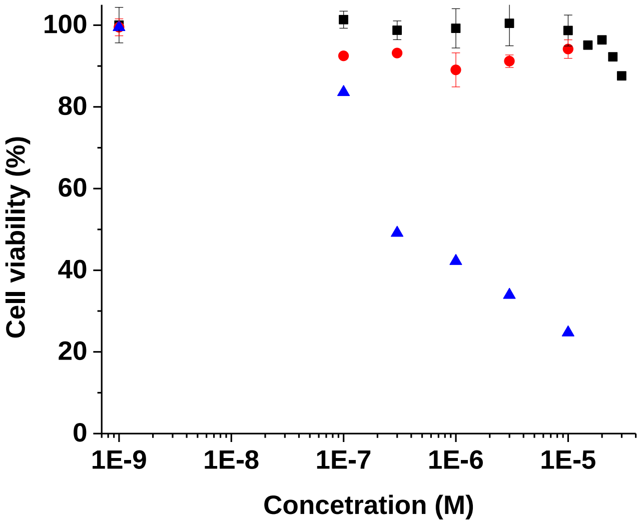

Figure S6. The effect of dye **8** and Doxorubicin (Dox) on cell viability. COS-7 cells were incubated with **8** for 3 h (red circle) or 24 h (black squares) or with Dox for 24 h (blue triangle).

References

1. Knorr, G.; Kozma, E.; Herner, A.; Lemke, E.A.; Kele, P. New Red-Emitting Tetrazine-Phenoxazine Fluorogenic Labels for Live-Cell Intracellular Bioorthogonal Labeling Schemes. *Chem. Eur. J.* **2016**, *22*, 8972–8979.
2. Kozma, E., Girona, G.E., Paci, G.; Lemke, E.A.; Kele, P. Bioorthogonal double-fluorogenic siliconrhodamine probes for intracellular super-resolution microscopy. *Chem. Commun.* **2017**, *53*, 6696–6699.
3. Kang, J.; Huo, F.; Yin, C. A novel fluorescent H2S probe based on tandem nucleophilic substitution/Cyclization reaction and its bioimaging. *Dyes and Pigments* **2017**, *146*, 287–292.
4. Knorr, G.; Kozma, E.; Schaart, J.M.; Németh, K.; Török, Gy.; Kele, P. Bioorthogonally Applicable Fluorogenic Cyanine-Tetrazines for No-Wash Super-Resolution Imaging. *Bioconjugate Chem.* **2018**, *29*, 1312–1318.
5. Wieczorek, A.; Werther, P.; Euchner, J.; Wombacher, R. Green- to far-red-emitting fluorogenic tetrazine probes - synthetic access and no-wash protein imaging inside living cells. *Chem. Sci.* **2017**, *8*, 1506–1510.
6. Meimetis, L. G.; Carlson, J. C.; Giedt, R. J.; Kohler, R. H.; Weissleder, R. Ultrafluorogenic coumarin-tetrazine probes for real-time biological imaging. *Angew. Chem. Int. Ed.* **2014**, *53*, 7531–7534
7. Egyed, A.; Kormos, A.; Söveges, B., Németh, K.; Kele,P. Biothogonally applicable, π-extended rhodamines for super-resolution microscopy imaging for intracellular proteins. *Bioorg. Med. Chem.* 2020, *28*, 115218.

NMR spectra

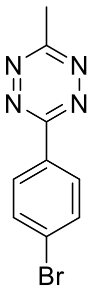

S1

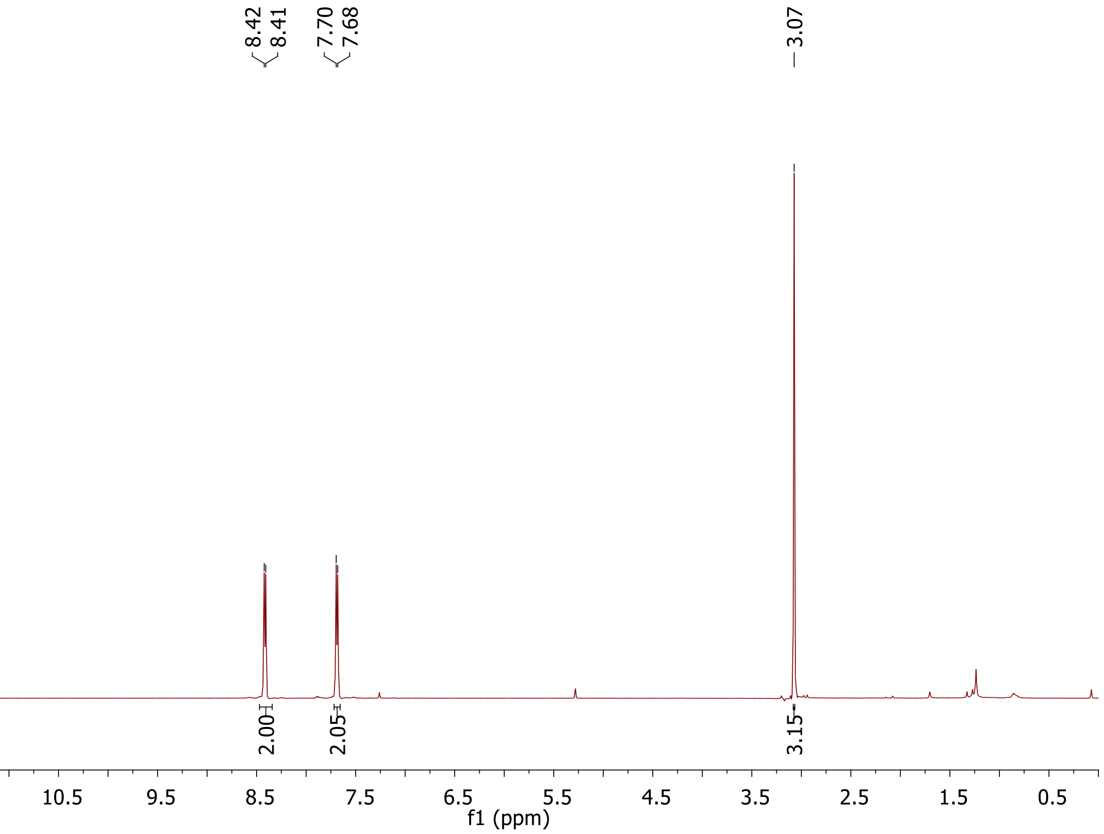

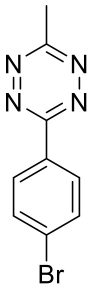

S1

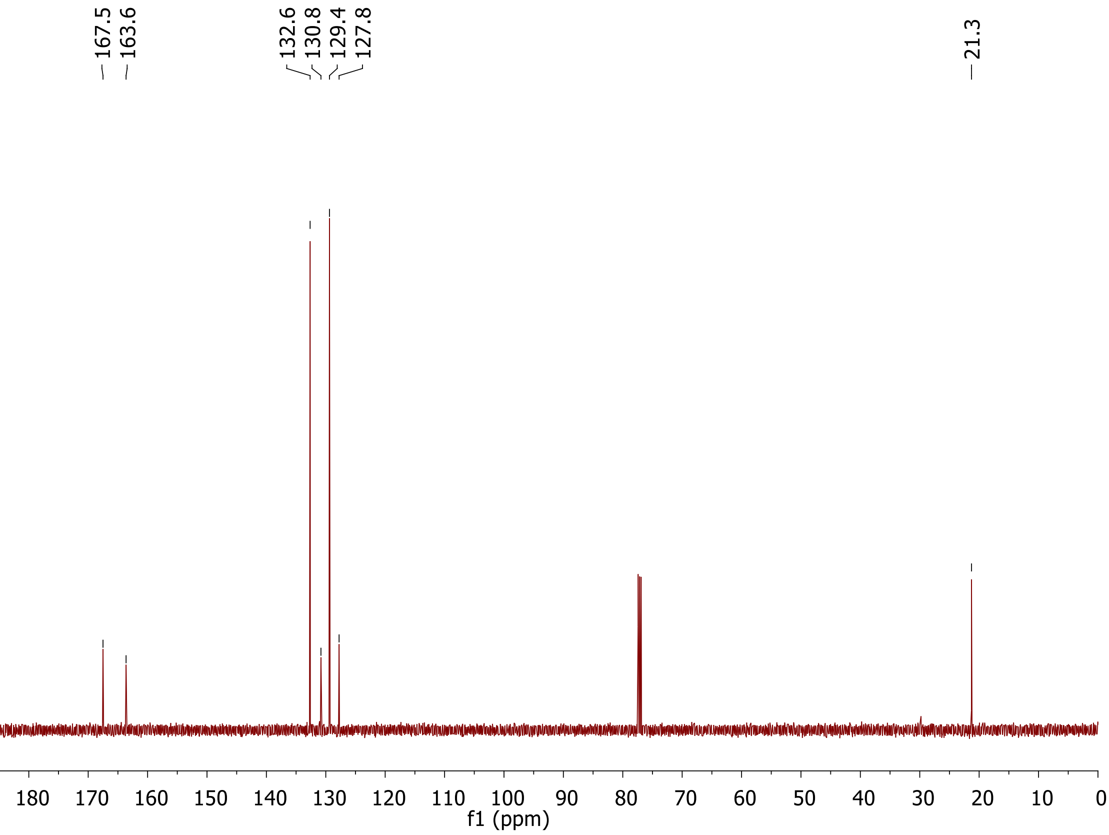

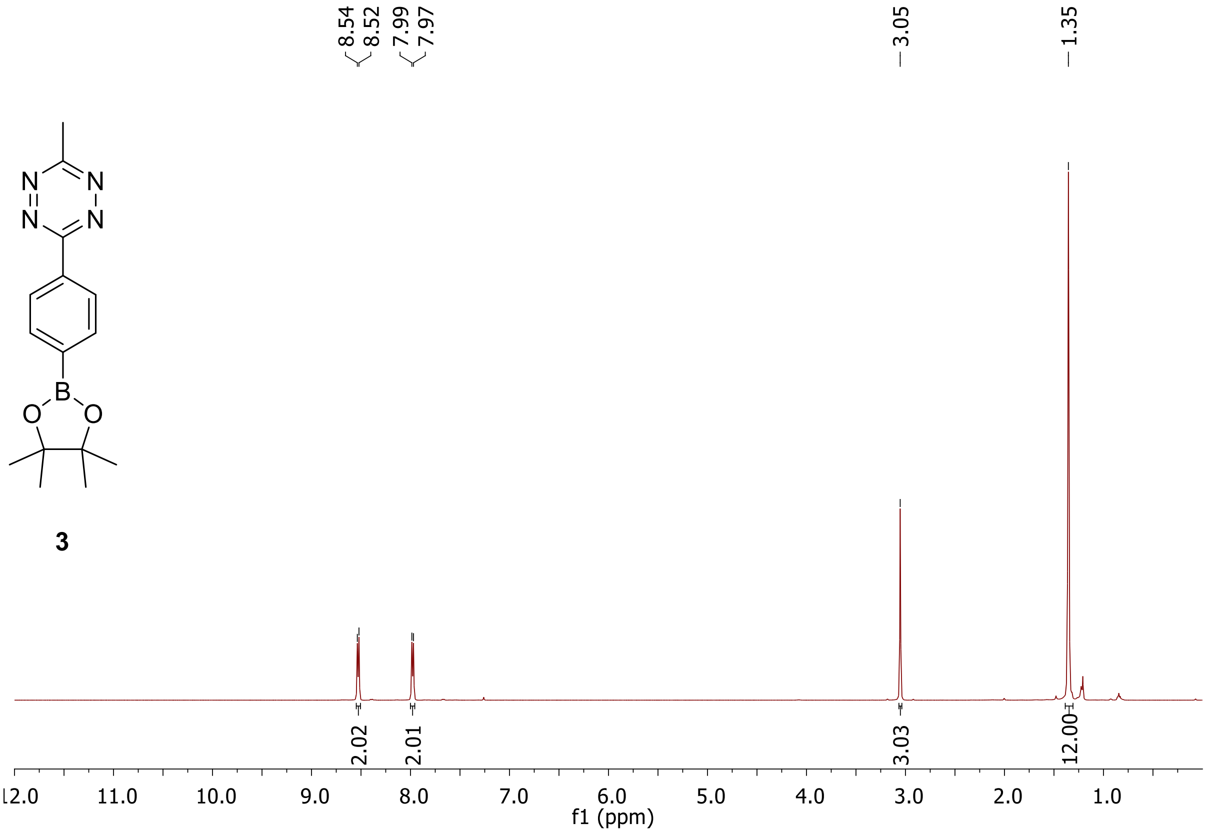
8.54
8.52
7.99
7.97
3.05
1.35
3
2.02
2.01
3.03
12.00
12.0 11.0 10.0 9.0 8.0 7.0 6.0 5.0 4.0 3.0 2.0 1.0
f1 (ppm)

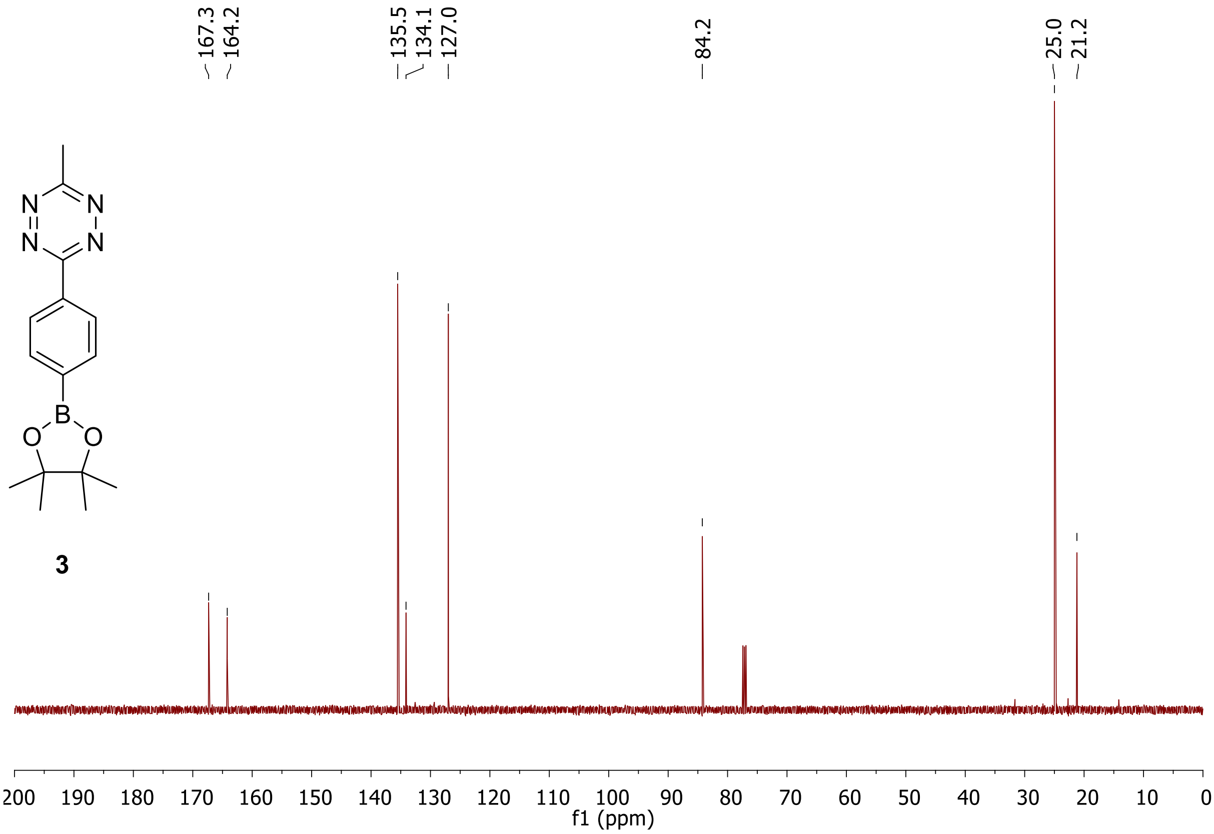
167.3
164.2
135.5
134.1
127.0
84.2
25.0
21.2
3
200 190 180 170 160 150 140 130 120 110 100 90 80 70 60 50 40 30 20 10 0
f1 (ppm)

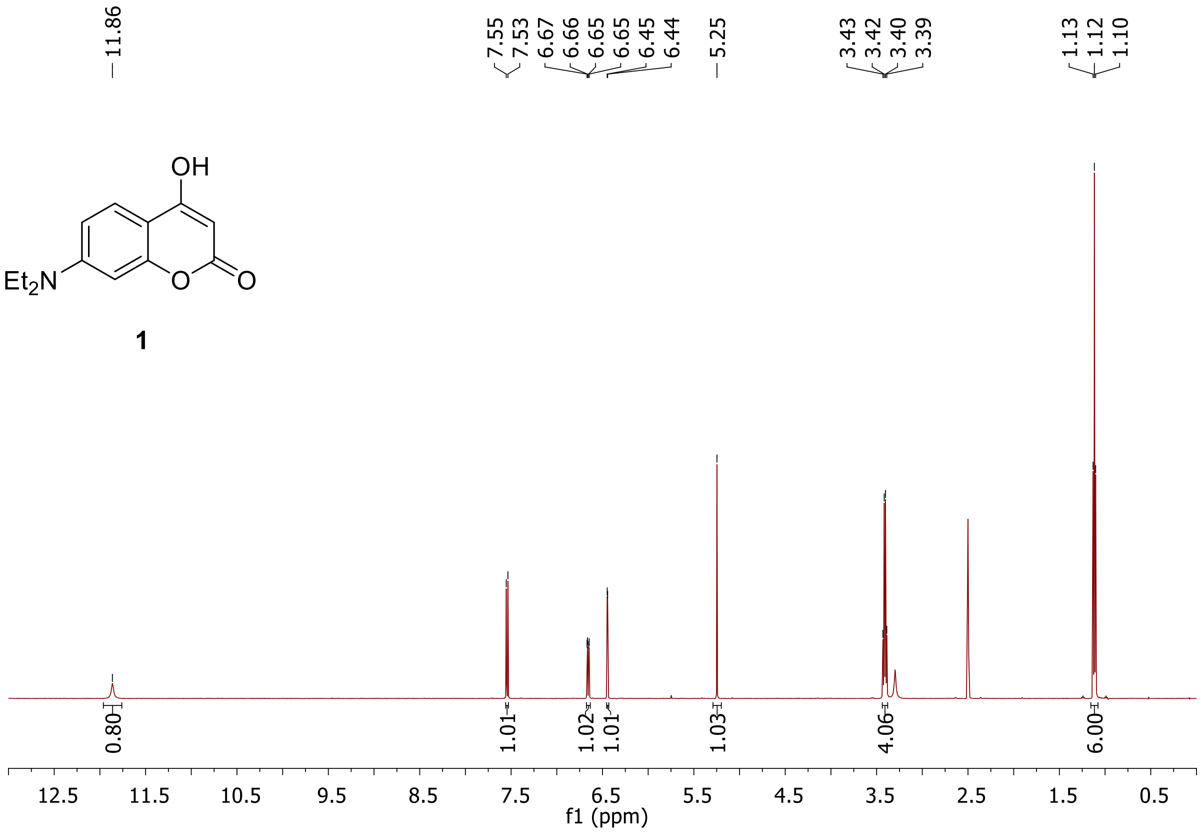
11.86
7.55
7.53
6.67
6.66
6.65
6.65
6.45
6.44
5.25
3.43
3.42
3.40
3.39
1.13
1.12
1.10
OH
Et2N
O
O
1
0.80
1.01
1.02
1.01
1.03
4.06
6.00
12.5 11.5 10.5 9.5 8.5 7.5 6.5 5.5 4.5 3.5 2.5 1.5 0.5
f1 (ppm)

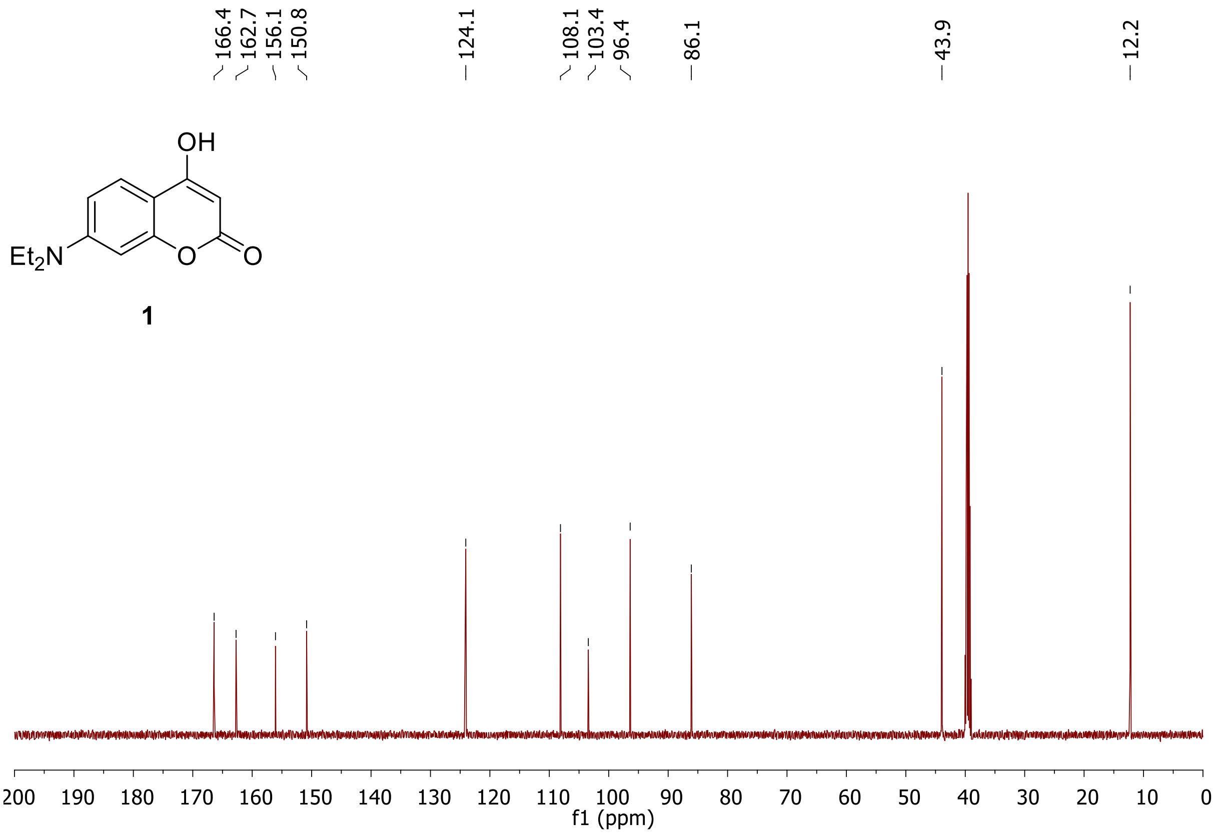
166.4
162.7
156.1
150.8
124.1
108.1
103.4
96.4
86.1
43.9
12.2
OH
Et2N
O
O
1
200 190 180 170 160 150 140 130 120 110 100 90 80 70 60 50 40 30 20 10 0
f1 (ppm)

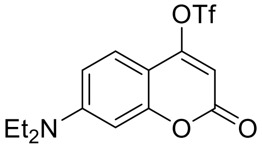
OTf
Et2N
O
O

3

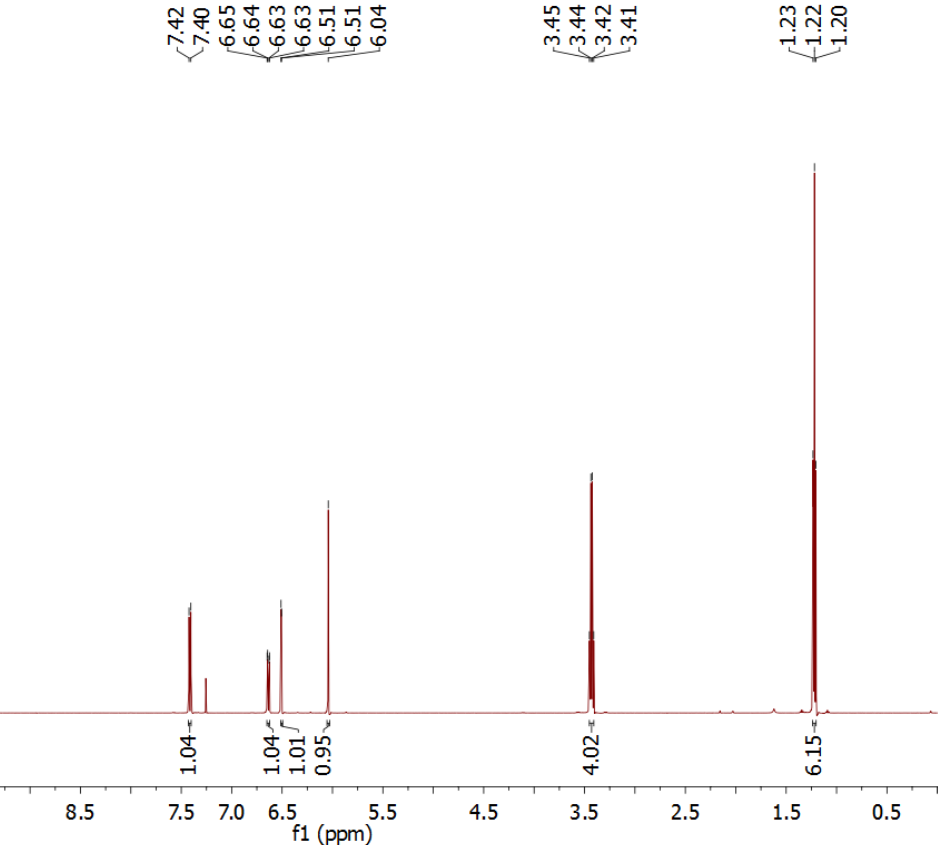
7.42
7.40
6.65
6.64
6.63
6.63
6.51
6.51
6.04
3.45
3.44
3.42
3.41
1.23
1.22
1.20
1.04
1.04
1.01
0.95
4.02
6.15
11.5
10.5
9.5
8.5
7.5
7.0
6.5
5.5
4.5
3.5
2.5
1.5
0.5
f1 (ppm)

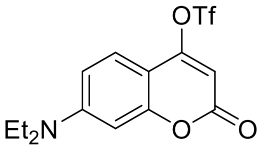
OTf
Et2N
O
O

3

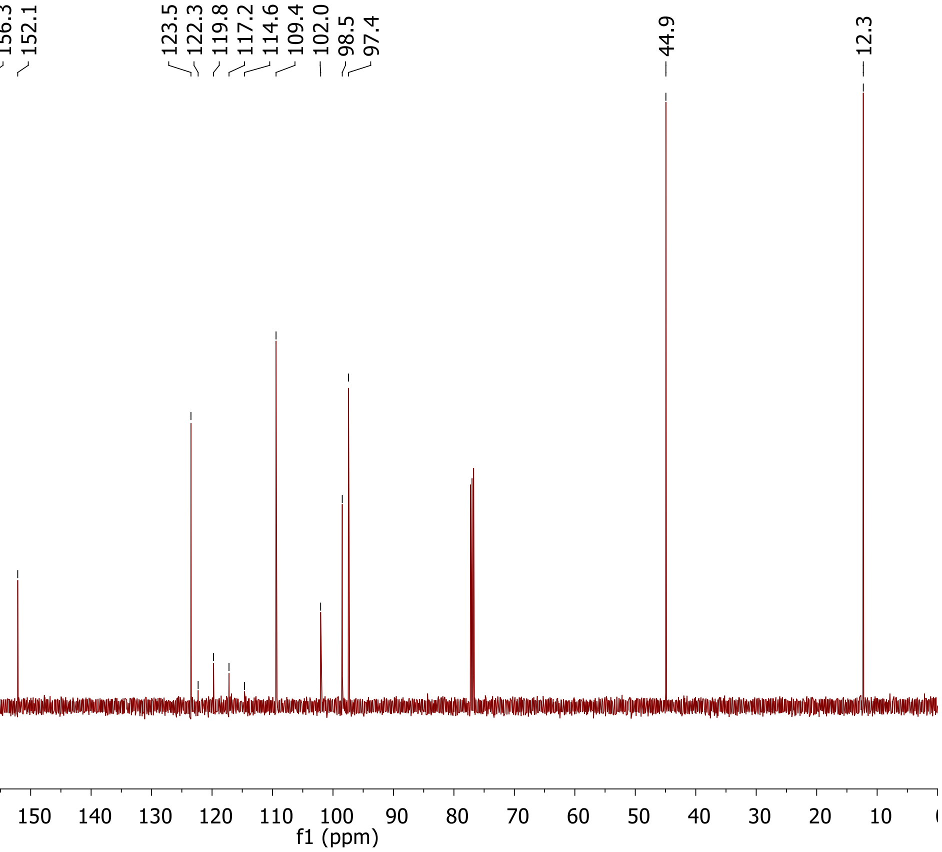
161.2
158.1
156.3
152.1
123.5
122.3
119.8
117.2
114.6
109.4
102.0
98.5
97.4
44.9
12.3
190
180
170
160
150
140
130
120
110
100
90
80
70
60
50
40
30
20
10
f1 (ppm)

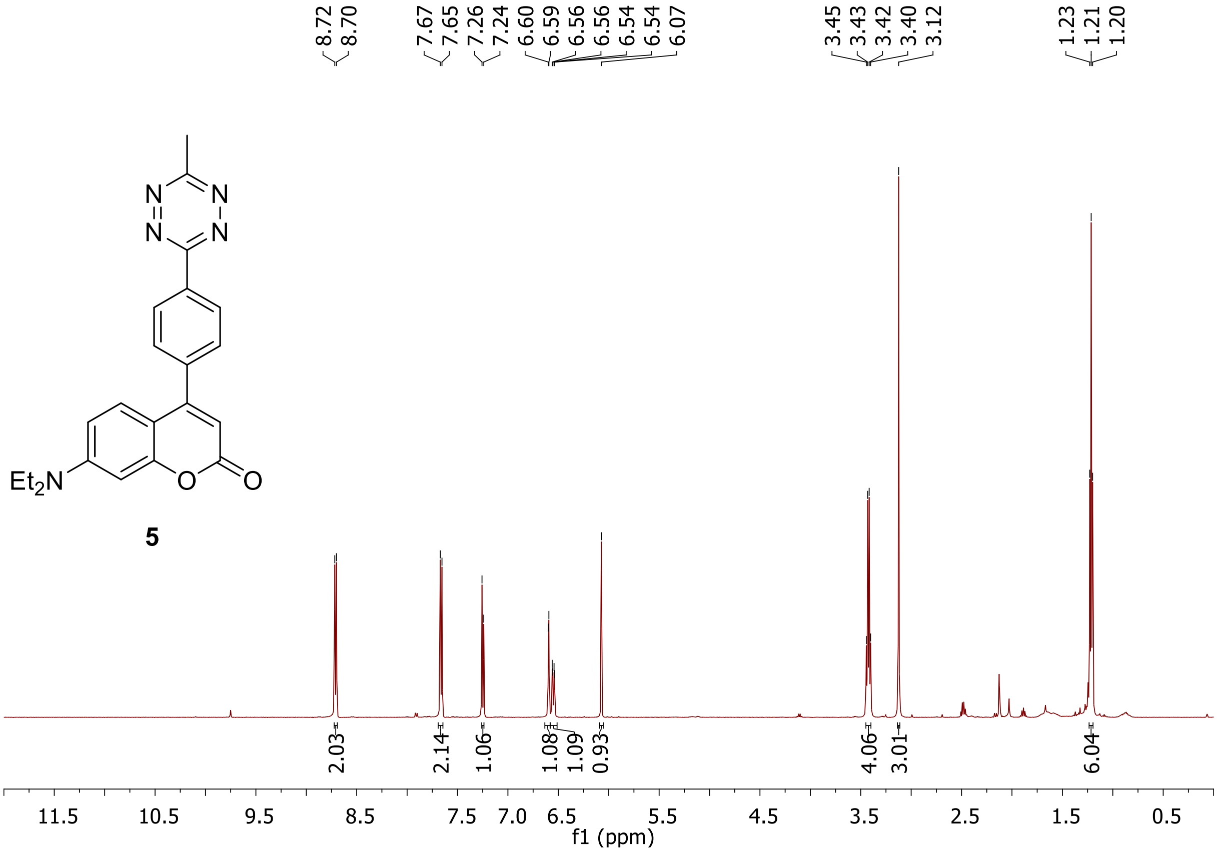

8.72
8.70
7.67
7.65
7.26
7.24
6.60
6.59
6.56
6.56
6.54
6.54
6.07
3.45
3.43
3.42
3.40
3.12
1.23
1.21
1.20
Et2N
O
O
N
N
N
N
5
2.03
2.14
1.06
1.08
1.09
0.93
4.06
3.01
6.04
11.5
10.5
9.5
8.5
7.5
7.0
6.5
5.5
4.5
3.5
2.5
1.5
0.5
f1 (ppm)

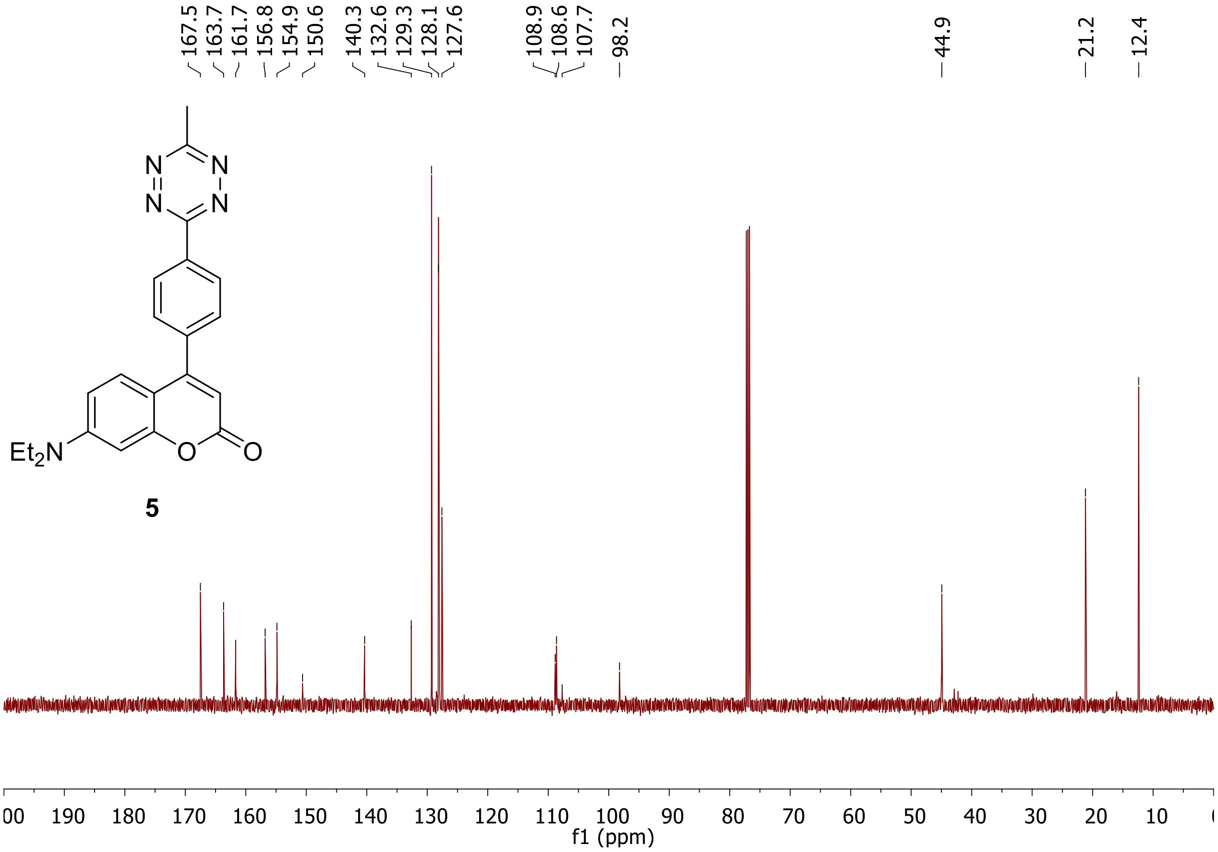

167.5
163.7
161.7
156.8
154.9
150.6
140.3
132.6
129.3
128.1
127.6
108.9
108.6
107.7
98.2
44.9
21.2
12.4
Et2N
O
O
N
N
N
N
5
00
190
180
170
160
150
140
130
120
110
100
90
80
70
60
50
40
30
20
10
f1 (ppm)

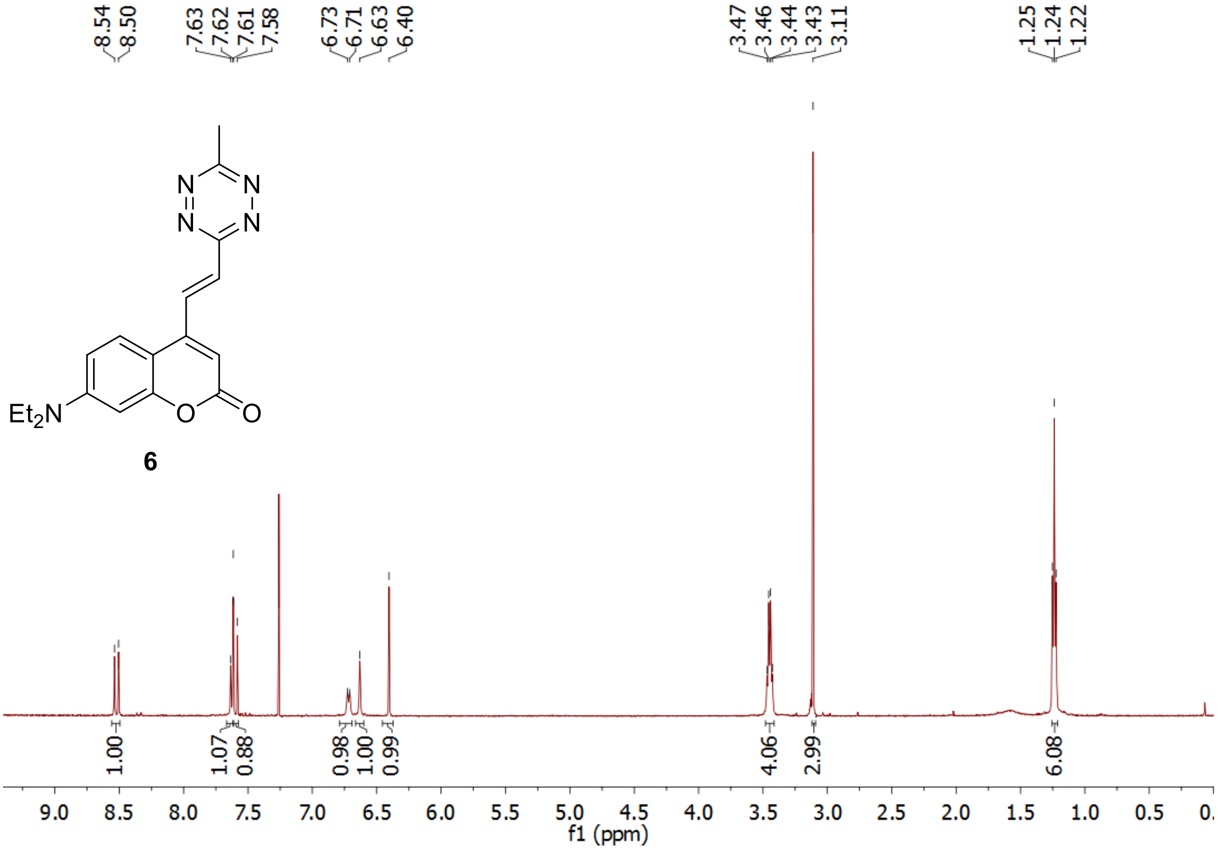
8.54
8.50
7.63
7.62
7.61
7.58
6.73
6.71
6.63
6.40
3.47
3.46
3.44
3.43
3.11
1.25
1.24
1.22
N
N
N
N
Et$_2$N
O
O
6
1.00
1.07
0.88
0.98
1.00
0.99
4.06
2.99
6.08
9.0 8.5 8.0 7.5 7.0 6.5 6.0 5.5 5.0 4.5 4.0 3.5 3.0 2.5 2.0 1.5 1.0 0.5 0.
f1 (ppm)

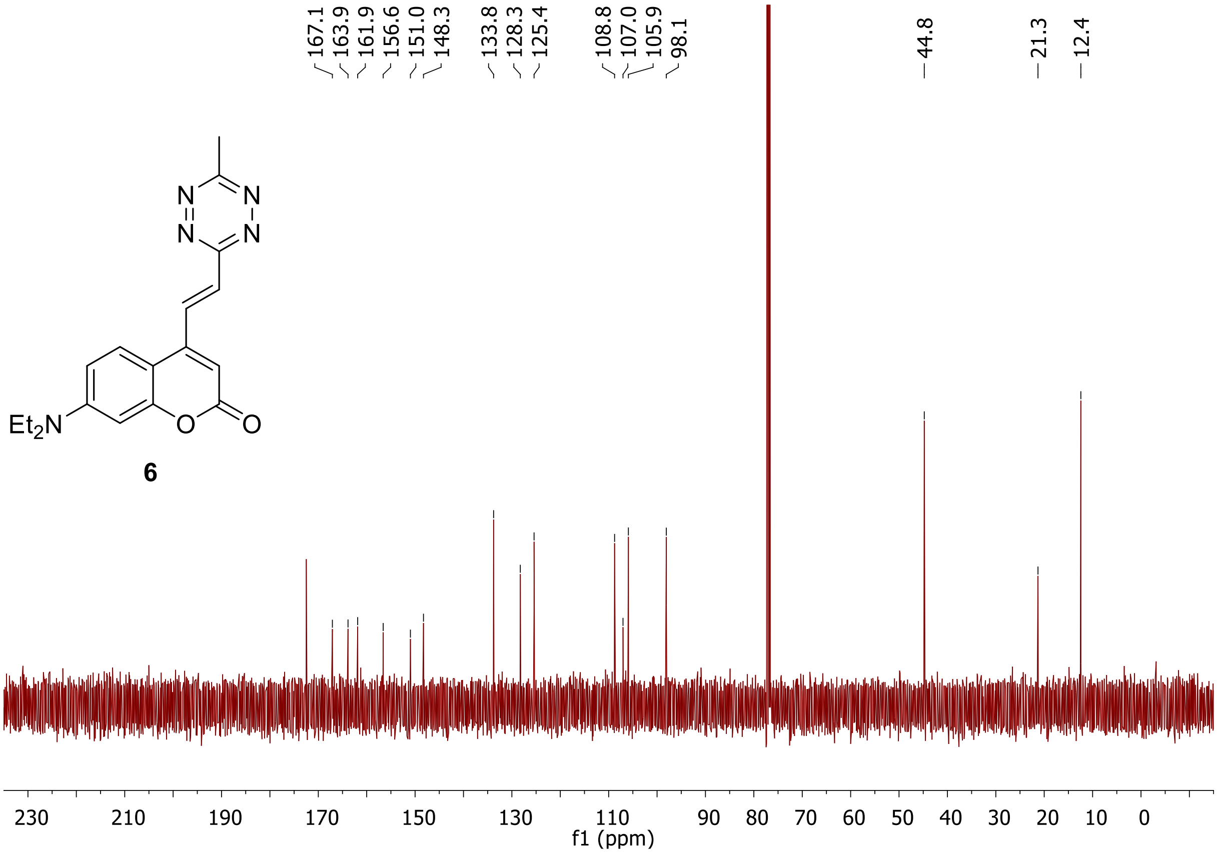
167.1
163.9
161.9
156.6
151.0
148.3
133.8
128.3
125.4
108.8
107.0
105.9
98.1
44.8
21.3
12.4
N
N
N
N
Et$_2$N
O
O
6
230 210 190 170 150 130 110 90 80 70 60 50 40 30 20 10 0
f1 (ppm)

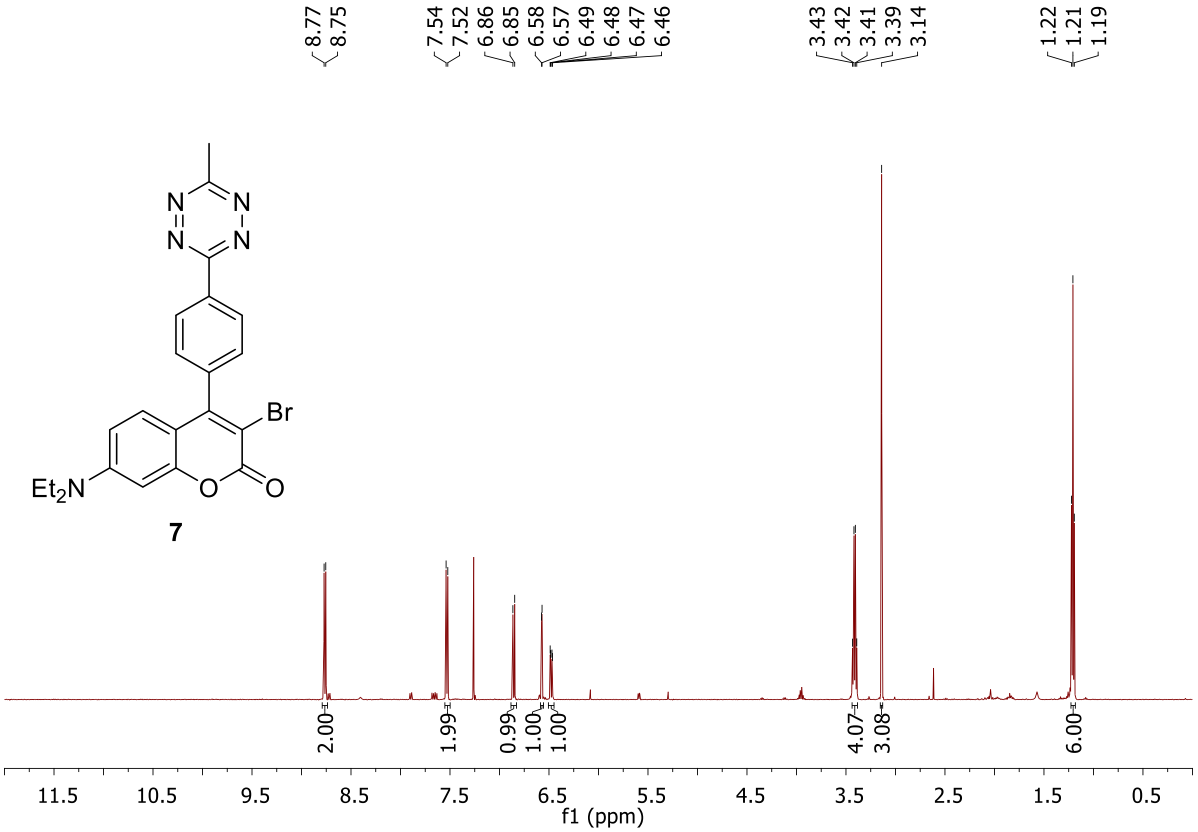
8.77
8.75
7.54
7.52
6.86
6.85
6.58
6.57
6.49
6.48
6.47
6.46
3.43
3.42
3.41
3.39
3.14
1.22
1.21
1.19
N
N
N
N
Br
Et_2N
O
O
7
2.00
1.99
0.99
1.00
1.00
4.07
3.08
6.00
11.5
10.5
9.5
8.5
7.5
6.5
5.5
4.5
3.5
2.5
1.5
0.5
f1 (ppm)

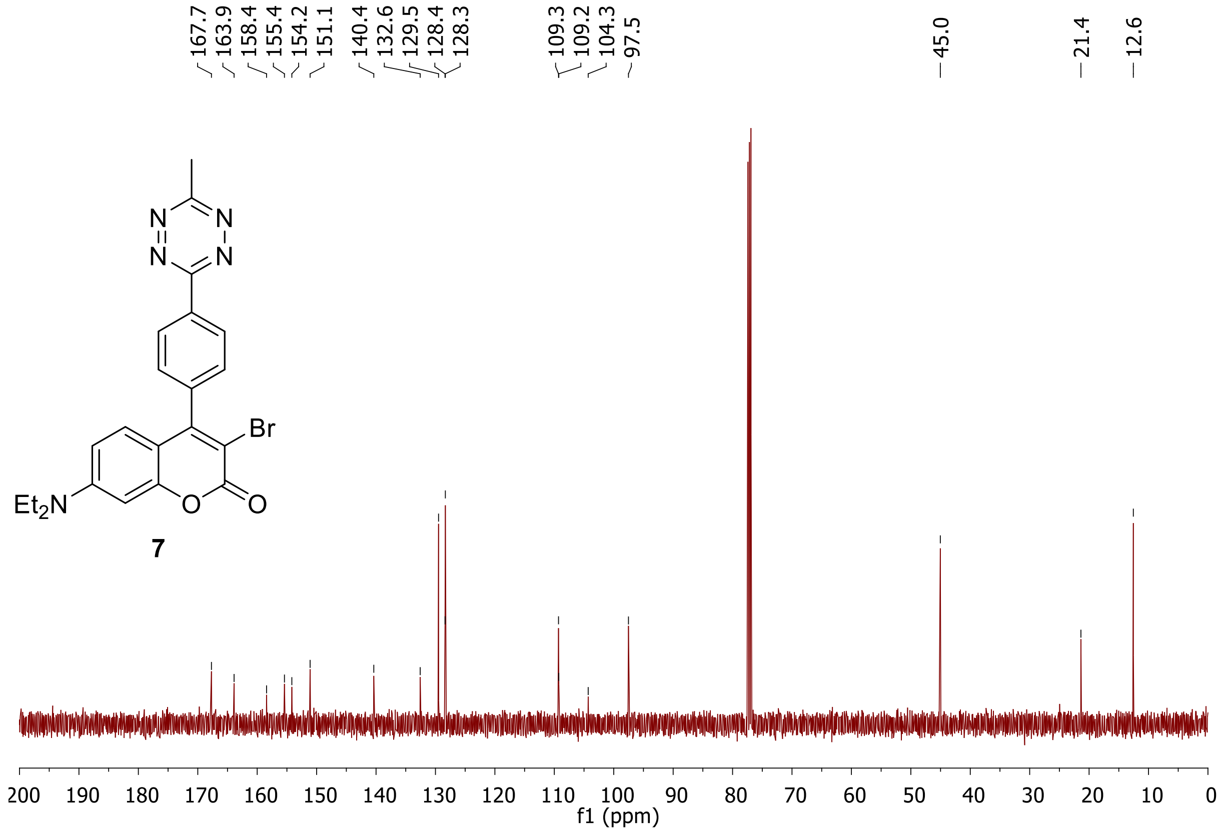
167.7
163.9
158.4
155.4
154.2
151.1
140.4
132.6
129.5
128.4
128.3
109.3
109.2
104.3
97.5
45.0
21.4
12.6
N
N
N
N
Br
Et_2N
O
O
7
200
190
180
170
160
150
140
130
120
110
100
90
80
70
60
50
40
30
20
10
0
f1 (ppm)

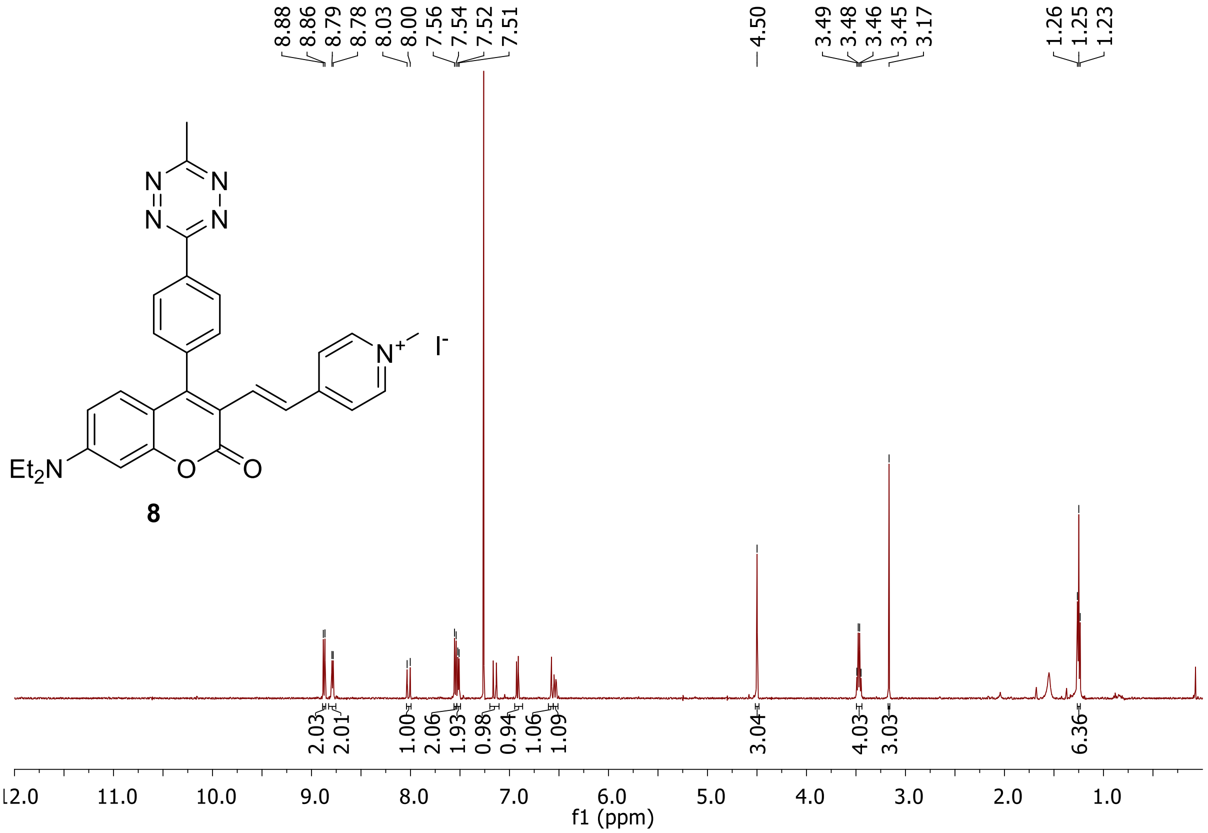
8.88
8.86
8.79
8.78
8.03
8.00
7.56
7.54
7.52
7.51
4.50
3.49
3.48
3.46
3.45
3.17
1.26
1.25
1.23
N
N
N
N
N^+
I^-
Et_2N
O
O
8
2.03
2.01
1.00
2.06
1.93
0.98
0.94
1.06
1.09
3.04
4.03
3.03
6.36
12.0
11.0
10.0
9.0
8.0
7.0
6.0
5.0
4.0
3.0
2.0
1.0
f1 (ppm)

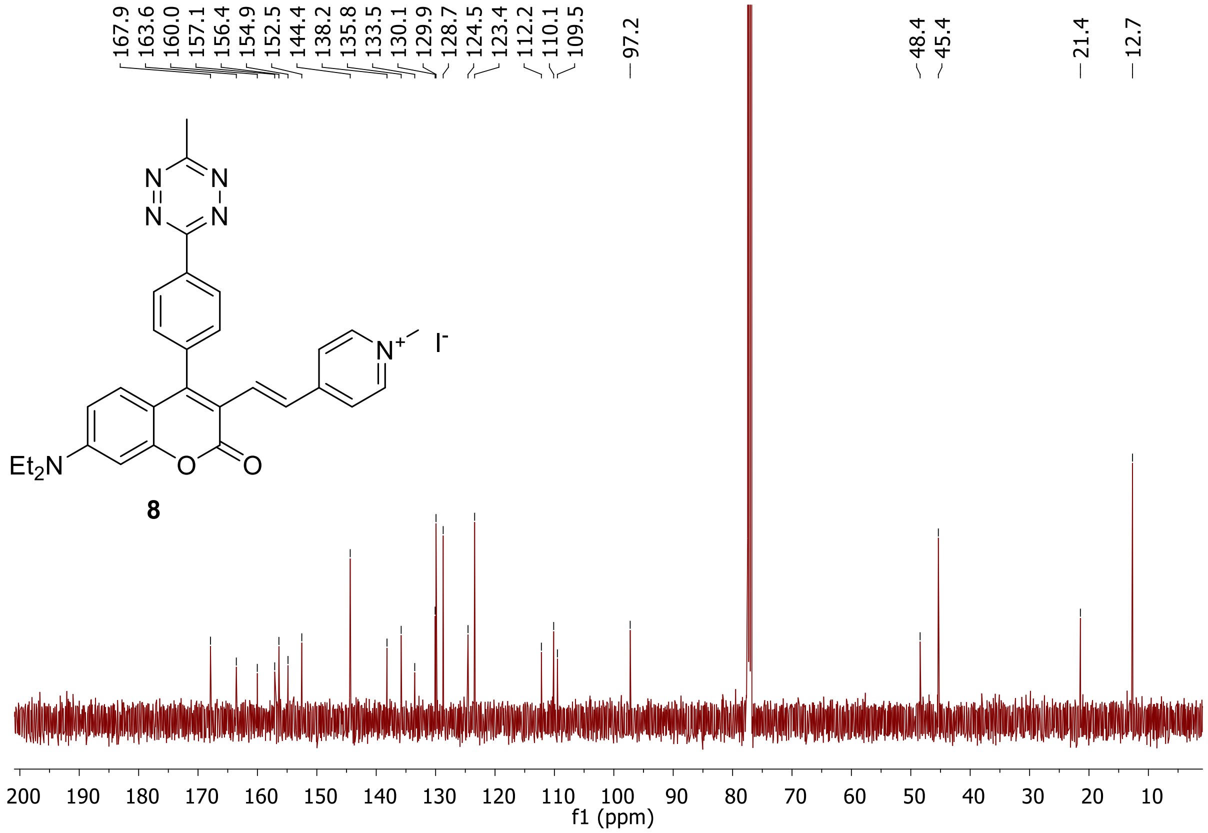
167.9
163.6
160.0
157.1
156.4
154.9
152.5
144.4
138.2
135.8
133.5
130.1
129.9
128.7
124.5
123.4
112.2
110.1
109.5
97.2
48.4
45.4
21.4
12.7
N
N
N
N
N^+
I^-
Et_2N
O
O
8
200
190
180
170
160
150
140
130
120
110
100
90
80
70
60
50
40
30
20
10
f1 (ppm)
